# *MEN1* is a regulator of alternative splicing and prevents R-loop-induced genome instability through suppression of RNA polymerase II elongation

**DOI:** 10.1093/nar/gkad548

**Published:** 2023-07-03

**Authors:** Bangming Jin, Jiamei Zhu, Ting Pan, Yunqiao Yang, Li Liang, Yuxia Zhou, Tuo Zhang, Yin Teng, Ziming Wang, Xuyan Wang, Qianting Tian, Bing Guo, Haiyang Li, Tengxiang Chen

**Affiliations:** Department of Physiology, School of Basic Medical Sciences, Guizhou Medical University, 550025 Guiyang, China; Department of Surgery, Affiliated Hospital of Guizhou Medical University, 550025 Guiyang, China; Transformation Engineering Research Center of Chronic Disease Diagnosis and Treatment, Guizhou Medical University, Guiyang, China; Guizhou Provincial Key Laboratory of Pathogenesis and Drug Research on Common Chronic Diseases, Guizhou Medical University, 550025 Guiyang, China; Department of Physiology, School of Basic Medical Sciences, Guizhou Medical University, 550025 Guiyang, China; Transformation Engineering Research Center of Chronic Disease Diagnosis and Treatment, Guizhou Medical University, Guiyang, China; Guizhou Provincial Key Laboratory of Pathogenesis and Drug Research on Common Chronic Diseases, Guizhou Medical University, 550025 Guiyang, China; Department of Physiology, School of Basic Medical Sciences, Guizhou Medical University, 550025 Guiyang, China; Transformation Engineering Research Center of Chronic Disease Diagnosis and Treatment, Guizhou Medical University, Guiyang, China; Guizhou Provincial Key Laboratory of Pathogenesis and Drug Research on Common Chronic Diseases, Guizhou Medical University, 550025 Guiyang, China; Department of Physiology, School of Basic Medical Sciences, Guizhou Medical University, 550025 Guiyang, China; Department of Surgery, Affiliated Hospital of Guizhou Medical University, 550025 Guiyang, China; Transformation Engineering Research Center of Chronic Disease Diagnosis and Treatment, Guizhou Medical University, Guiyang, China; Guizhou Provincial Key Laboratory of Pathogenesis and Drug Research on Common Chronic Diseases, Guizhou Medical University, 550025 Guiyang, China; Department of Physiology, School of Basic Medical Sciences, Guizhou Medical University, 550025 Guiyang, China; Transformation Engineering Research Center of Chronic Disease Diagnosis and Treatment, Guizhou Medical University, Guiyang, China; Guizhou Provincial Key Laboratory of Pathogenesis and Drug Research on Common Chronic Diseases, Guizhou Medical University, 550025 Guiyang, China; Department of Physiology, School of Basic Medical Sciences, Guizhou Medical University, 550025 Guiyang, China; Transformation Engineering Research Center of Chronic Disease Diagnosis and Treatment, Guizhou Medical University, Guiyang, China; Guizhou Provincial Key Laboratory of Pathogenesis and Drug Research on Common Chronic Diseases, Guizhou Medical University, 550025 Guiyang, China; Department of Physiology, School of Basic Medical Sciences, Guizhou Medical University, 550025 Guiyang, China; Transformation Engineering Research Center of Chronic Disease Diagnosis and Treatment, Guizhou Medical University, Guiyang, China; Guizhou Provincial Key Laboratory of Pathogenesis and Drug Research on Common Chronic Diseases, Guizhou Medical University, 550025 Guiyang, China; Department of Surgery, Affiliated Hospital of Guizhou Medical University, 550025 Guiyang, China; Guizhou Institute of Precision Medicine, Affiliated Hospital of Guizhou Medical University, 550025 Guiyang, China; Department of Physiology, School of Basic Medical Sciences, Guizhou Medical University, 550025 Guiyang, China; Transformation Engineering Research Center of Chronic Disease Diagnosis and Treatment, Guizhou Medical University, Guiyang, China; Guizhou Provincial Key Laboratory of Pathogenesis and Drug Research on Common Chronic Diseases, Guizhou Medical University, 550025 Guiyang, China; Department of Physiology, School of Basic Medical Sciences, Guizhou Medical University, 550025 Guiyang, China; Transformation Engineering Research Center of Chronic Disease Diagnosis and Treatment, Guizhou Medical University, Guiyang, China; Guizhou Provincial Key Laboratory of Pathogenesis and Drug Research on Common Chronic Diseases, Guizhou Medical University, 550025 Guiyang, China; Transformation Engineering Research Center of Chronic Disease Diagnosis and Treatment, Guizhou Medical University, Guiyang, China; Guizhou Institute of Precision Medicine, Affiliated Hospital of Guizhou Medical University, 550025 Guiyang, China; Department of Physiology, School of Basic Medical Sciences, Guizhou Medical University, 550025 Guiyang, China; Transformation Engineering Research Center of Chronic Disease Diagnosis and Treatment, Guizhou Medical University, Guiyang, China; Guizhou Provincial Key Laboratory of Pathogenesis and Drug Research on Common Chronic Diseases, Guizhou Medical University, 550025 Guiyang, China; Department of Surgery, Affiliated Hospital of Guizhou Medical University, 550025 Guiyang, China; Guizhou Institute of Precision Medicine, Affiliated Hospital of Guizhou Medical University, 550025 Guiyang, China; Department of Physiology, School of Basic Medical Sciences, Guizhou Medical University, 550025 Guiyang, China; Department of Surgery, Affiliated Hospital of Guizhou Medical University, 550025 Guiyang, China; Transformation Engineering Research Center of Chronic Disease Diagnosis and Treatment, Guizhou Medical University, Guiyang, China; Guizhou Provincial Key Laboratory of Pathogenesis and Drug Research on Common Chronic Diseases, Guizhou Medical University, 550025 Guiyang, China

## Abstract

The fidelity of alternative splicing (AS) patterns is essential for growth development and cell fate determination. However, the scope of the molecular switches that regulate AS remains largely unexplored. Here we show that *MEN1* is a previously unknown splicing regulatory factor. *MEN1* deletion resulted in reprogramming of AS patterns in mouse lung tissue and human lung cancer cells, suggesting that *MEN1* has a general function in regulating alternative precursor mRNA splicing. *MEN1* altered exon skipping and the abundance of mRNA splicing isoforms of certain genes with suboptimal splice sites. Chromatin immunoprecipitation and chromosome walking assays revealed that *MEN1* favored the accumulation of RNA polymerase II (Pol II) in regions encoding variant exons. Our data suggest that *MEN1* regulates AS by slowing the Pol II elongation rate and that defects in these processes trigger R-loop formation, DNA damage accumulation and genome instability. Furthermore, we identified 28 *MEN1*-regulated exon-skipping events in lung cancer cells that were closely correlated with survival in patients with lung adenocarcinoma, and *MEN1* deficiency sensitized lung cancer cells to splicing inhibitors. Collectively, these findings led to the identification of a novel biological role for menin in maintaining AS homeostasis and link this role to the regulation of cancer cell behavior.

## Graphical Abstract

**Graphical Abstract:**
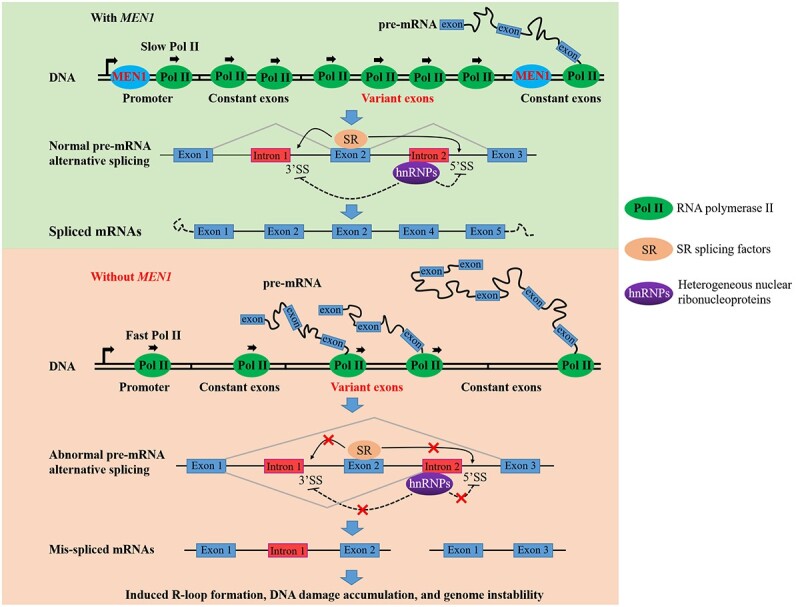

## INTRODUCTION

Alternative splicing (AS) in eukaryotes is a critical mechanism regulating post-transcriptional gene expression in eukaryotes. In this controlled process, precursor mRNA (pre-mRNA) is differentially spliced, which leads to the expression of several mRNA variants derived from a single gene (1). The discovery of AS led to the deciphering of how the metazoan proteome can come to possess different biological functions and tremendous complexity from a small number of genes. Nearly 90% of human pre-mRNAs undergo AS, generating mRNA isoforms that tend to be differentially expressed across distinct cells or tissues and developmental stages (2). The AS process is highly controlled through a variety of mechanisms, including the interaction of splicing factors with *cis*-acting elements in the pre-mRNA sequence, the rate and pausing of RNA polymerase II (Pol II) elongation and epigenetic modification of template chromatin (3). Dysregulation in the processing of alternatively spliced pre-mRNA has been closely linked to various human diseases, especially cancers. A comprehensive analysis revealed that nearly all cancer tissues exhibit abnormal AS profiles relative to their healthy tissue counterparts (4). An increasing number of studies have shown that tumor-specific splicing variants participate in the regulation of cellular processes, including proliferation (5), invasion (6,7), apoptosis (8), drug resistance (9) and metabolism (10). Thus, identifying tumor-specific AS and elucidating oncogenic mechanisms provide new opportunities for the development of cancer therapeutics.

Alternative pre-mRNA splicing depends on the precise recognition of splice sites and selective intron excision, processes that are mediated by the spliceosome, a dynamic ribonucleoprotein (RNP) complex consisting of five small nuclear RNAs (snRNAs; U1, U2, U4, U5 and U6) and a wide variety of proteins (11). The U1 and U2 small nuclear RNPs (snRNPs) are critical for the identification of splice sites, facilitating the recruitment of the U4/U5/U6 snRNP complex and various non-snRNPs. When U1/U4 snRNPs are subsequently displaced, the conformational changes and two splicing transesterification reactions are mediated through a catalytically active spliceosome consisting of U2, U5 and U6 snRNPs (12). Altered expression or disorderly assembly of spliceosome components can lead to pathological changes in AS in response to external damage. DNA damage has been proposed to impact the choice of splice site by disrupting spliceosome mobilization (13), and abnormal AS induced by DNA damage has been attributed to changes in the Pol II elongation rate or disrupted interactions between Pol II and splicing factors (14). Dysregulated recruitment of splicing factors to Pol II triggers DNA double-strand breaks (DSBs) (15). When aberrant regulators of splicing hinder the repair of DNA damage, carcinogenesis and tumor progression can result (16). Observations of aberrant AS processes and human diseases, once rare, have increased, yet understanding of the molecular mechanisms and biological significance of AS regulation in tumorigenesis remains in its infancy.

The tumor suppressor gene *MEN1* (*Men1* in mice) encodes the nuclear protein menin whose inactivation causes the development of multiple endocrine neoplasia type 1 (MEN1) (17,18). Functional studies have shown that *MEN1* is extensively involved in the regulation of genome stability (19,20), the DNA damage response (DDR) (21) and multiple cellular behaviors (22). Menin binds a variety of DNA structures, including Y-structures, branched structures and four-way junction structures, in a sequence-independent manner (23). Mechanistic analyses have demonstrated that menin is a bona fide transcription factor that binds promoter regions to activate the transcription of multiple genes (22); in fact, transcription factors recruited to promoter sequences influence the AS process (24). In line with its potential role in RNA splicing, menin localizes to SRSF2 nuclear speckles (25), which are involved in the regulation of alternative and constitutive splicing (26–28). These observations suggest a connection (that remains mechanistically obscure) between *MEN1* and the pre-mRNA splicing process.

Here, we report a previously unappreciated function of *MEN1* in regulating alternative pre-mRNA splicing. *MEN1*-regulated alternatively spliced genes contain weak 5′ or 3′ splice sites. In this study, mechanistically speaking, *MEN1* altered exon skipping and the abundance of splicing isoforms by slowing the rate of Pol II elongation. *MEN1* deletion led to R-loop formation and DNA damage accumulation, thereby inducing genome instability. Our results suggest that splicing inhibitors may be therapeutically beneficial for *MEN1*-inactivated cancers.

## MATERIALS AND METHODS

### Cell culture

NCI-H460, NCI-H446, A549 and HEK-293T cells were from the American Type Culture Collection (ATCC). Wild-type (WT) *Men1* (*Men1*^f/f^) and *Men1* knockout (KO) (*Men1*^Δ/Δ^) mouse embryonic fibroblasts (MEFs) were described previously (21). A549 and HEK-293T cells were maintained in Dulbecco's modified Eagle's medium (DMEM; Gibco) supplemented with 10% fetal bovine serum (FBS; Biological Industries) and 1% penicillin/streptomycin (P1400, Solarbio); NCI-H460 and NCI-H446 cells were maintained in RPMI-1640 (Gibco) supplemented with 10% FBS and 1% penicillin/streptomycin. All cell lines were cultured at 37°C with 5% CO_2_. The absence of mycoplasma contamination was identified in cultured cells using the Mycoplasma Stain Assay Kit (C0296, Beyotime).

### Generation of *men1* knockout mice

Mice with whole-body expression of *Ubc-Cre* were crossed with mice harboring floxed alleles of *Men1* to obtain conditional tamoxifen (TAM)-inducible *Men1*^flox/flox^ (*Men1*^f/f^);*Ubc-Cre* mice. Homozygous floxed *Men1*^f/f^ mice without *Cre* were used as controls. TAM was dissolved in corn oil containing 10% ethanol. At 4 weeks of age, the *Men1*^f/f^;*Ubc-Cre* and *Men1*^f/f^ mice received intraperitoneal (i.p.) administration of 100 mg/kg TAM (T5648-1G, Sigma) once a day for 5 days to generate *Men1* KO (*Men1*^Δ/Δ^) mice. Polymerase chain reaction (PCR) was used to verify the genotype. Mice were housed under standard conditions with a light/dark cycle of 12 h and free access to food and water. Animal experiments were performed in accordance with animal welfare and American Veterinary Medical Association (AVMA) Guidelines for the Euthanasia of Animals (2020), and the procedures were approved by the Institutional Animal Care Committee of Guizhou Medical University (no. 2100027).

### RNA sequencing and ASE analysis

RNA-seq was performed by Wuhan IGENEBOOK Bio-Tech Co., Ltd. Total RNA was extracted from lung tissue from 4-month-old *Men1*^f/f^ and *Men1*^Δ/Δ^ mice (*n* = 3 biological replicates per group) or *MEN1*-WT and *MEN1*-KO NCI-H460 cells (*n* = 2 biological replicates per group) using an RNAprep Pure Kit (DP432, Tiangen) following the manufacturer's instructions. RNA was assessed for quality and yield with a Qsep1 instrument (BiOptic). A 1 μg aliquot of total RNA was purified with poly(A) oligo-attached magnetic beads and fragmented to ∼150 nt in size, and cDNA was synthesized with random hexamer primers. RNA-seq was performed on an Illumina NovaSeq6000 platform with an average depth of ∼30 million, 150 nucleotide paired-end reads per sample. With base calling, raw image data files were converted into sequenced reads, referred to as raw data or raw reads. The adapter and low-quality reads were filtered in cutadapter (version 1.11) to generate clean reads. The clean reads were aligned to the mm9 mouse or GRCh38 human reference genome with STAR (version 3.2.5) (29). Transcripts were assembled in StringTie (version 2.0.4) (30), followed by estimates of raw gene counts in featureCounts (version v1.6.0) (31) and FPKM (fragments per kilobase of transcript per million mapped reads) normalization (32). Differentially expressed genes (DEGs) were identified with DESeq2 with a filter threshold of adjusted *P* < 0.05 and fold change ≥ 1.5.

Next, we conducted AS analysis using multivariate analysis of transcript splicing (rMATS) (version 3.2.5) (29). We used the sorted BAM files generated by STAR to run rMATS using default unpaired procedures. To identify significant differential splicing events, we set the following cut-offs: false discovery rate (FDR) < 0.05, |Δ percent spliced-in (ΔPSI)| ≥ 0.1 and average junction reads per event of each replicate ≥ 20. Modeling alternative junction inclusion quantification (MAJIQ) and Voila (https://biociphers.bitbucket.io/majiq/index.html) were used to determine, quantify and visualize local splicing variants (LSVs) from the RNA-seq data (33). Briefly, the MAJIQ build tool used the BAM alignment files from STAR along with a gene annotation file to define splice diagrams, and MAJIQ PSI and ΔPSI were used to calculate relative levels (PSI) of LSVs and changes in relative LSV abundance (ΔPSI) between the two conditions. For the MAJIQ data analysis, Voila TSV software was used to create a tab-delimited text file and then analyze specific LSVs or spliced genes of interest. Gene Ontology (GO) or Kyoto Encyclopedia of Genes and Genomes (KEGG) enrichment analysis was performed on the AS events (ASEs) identified as having differential splicing changes using clusterProfiler.

### Real-time quantitative PCR (qPCR) and reverse–transcription PCR (RT–PCR)

RNA preparation was conducted with TRIzol reagent (10606ES60, Yeasen), and cDNA synthesis was performed according to the instructions on the reverse transcription kit (11141ES60, Yeasen). qPCR was carried out with TB Green® Premix Ex Taq™ (RR820A, TaKaRa) on a CFX Connect™ Real-Time System (CFXConne, Bio-Rad). qPCRs were performed in at least three independent experiments, and relative gene expression was quantified based on the 2^−ΔΔCt^ method with reference genes as a normalization indicated control. For RT–PCR assays, based on ASE analysis, primers for detecting exon skipping were designed in Primer Premier 5 for validation of RNA splicing. RT–PCR was performed with the following program: 95°C for 2 min, 36 cycles of denaturation at 95°C for 30 s, annealing at 56°C for 30 s and elongation at 72°C for 1 min, and a final elongation temperature of 72°C for 5 min and 4°C holding temperature. PCR products were run in 2% agarose gels stained with 0.1‰ ethidium bromide (EB) dye (C14141868, Macklin). Bands were visualized with a GleUV system (Baygene Biotech). Band intensities were quantified in ImageJ software.

### Chromatin immunoprecipitation (ChIP)-qPCR assay

ChIP assays were performed according to the instructions for the Simple ChIP Kit (9003S, CST). Briefly, 1 × 10^6^ cells were cross-linked with 1% (v/v) formaldehyde for 10 min at room temperature. Cell pellets were incubated in Buffer A for 10 min at 4°C. Pellet nuclei were collected by centrifugation at 2000 *g* and digested in Buffer B containing 25 U of micrococcal nuclease (MNase) (10011, CST) per immunoprecipitation (IP) for 20 min at 37°C, followed by pulsed ultrasonication to shear cellular DNA, and harvested by centrifugation at 12 000 *g* for 10 min. After quantification of chromatin DNA, equal amounts of chromatin were incubated overnight at 4°C with the specific antibodies or non-immune IgG as a negative control. Protein A/G magnetic beads were used to couple the immunoprecipitated complexes for 2 h at 4°C, and then these bound complexes were rinsed extensively with washing buffer. Next, DNA pulled down by the antibodies was purified on spin columns, and purified DNA was quantified by qPCR with TB Green Premix Ex Taq™ on a CFX Connect Real-Time System. Values were calculated relative to the input and enriched relative to the signal obtained for IgG (set to 1). The following primary antibodies were adopted: menin (ab31902, Abcam, 6 μg/IP), histone H3 (4620, CST, 3 μg/IP), Pol II (ab264350, Abcam, 8 μg/IP), p-Pol II (Ser2) (ab5095, Abcam, 5 μg/IP), p-Pol II (Ser5) (2629, CST, 5 μg/IP), U2AF65 (sc-53942, Santa Cruz, 5 μg/IP) and SNRPA (10212–1-AP, Proteintech, 5 μg/IP).

### DNA–RNA immunoprecipitation (DRIP) assay

DRIP assays were performed according to a previously established protocol (34). Briefly, *MEN1*-WT and *MEN1*-KO NCI-H460 cells were rinsed three times in phosphate-buffered saline (PBS), resuspended in TE buffer (5 mM EDTA and 50 mM Tris–HCl, pH 8.0) containing 5 μl of proteinase K, and lysed overnight at 37°C by the addition of sodium dodecylsulfate (SDS) to a final concentration of 0.5%. Genomic DNA was extracted with the phenol/chloroform procedure and enzymatically digested overnight at 37°C with a subset of restriction enzymes (20 U of EcoRI, 20 U of HindIII, 20 U of XbaI, 25 U of Sspl and 10 U of BsrGI) in buffer. Digested DNA was purified by standard phenol/chloroform extraction and ethanol precipitation as described previously. As a negative control, digested DNA was incubated with 10 U/ml RNase H (New England Biolabs) overnight at 37°C. For DRIP experiments, 10 μg of digested DNA was immunoprecipitated with 10 μg of S9.6 antibody (MABE1095, Millipore) overnight at 4°C in 1× DRIP buffer (100 mM NaH_2_PO_4_, 1.4 M NaCl and 0.5% Triton X-100). A 20 μl aliquot of protein G Dynabeads was pre-washed with 1× DRIP buffer for 20 min at room temperature, and then these Dynabeads were added to couple the IP complexes for 2 h at 4°C. Next, immunoprecipitates were washed three times with 1× DRIP buffer at room temperature. After the last wash, DNA was eluted by addition of elution buffer containing 50 mM Tris–HCl (pH 8.0), 10 mM EDTA and 0.5% SDS, and incubated at 65°C for 45 min. Finally, DNA was purified using a Universal DNA Purification Kit (DP214-03, Tiangen) according to the manufacturer's instructions. Locus-specific DRIP signals were analyzed by qPCR, and the IP rate was expressed as the input percentage. Relative values with respect to *MEN1*-WT cells without RNase H were also determined and plotted.

### Chromatin-associated snRNA (caRNA) isolation

We prepared caRNAs by modifying a method described in previous reports for the isolation of ternary complex-associated nascent RNA (35). Briefly, cell pellets were resuspended in a lysis buffer containing 20 mM HEPES (pH 7.5), 10 mM KCl, 250 mM sucrose, 5 mM MgCl_2_, 1 mM EGTA, 1 mM phenylmetylsulfonyl fluoride (PMSF), 1× phosphatase inhibitor, 1 μl/ml RNase inhibitor (R8061, Solarbio) and 1× protease inhibitor cocktail (P6730, Solarbio), and lysed for 10 min at 4°C by the addition of digitonin (11024–24-1, Sigma) to a final concentration of 200 μg/ml. Nuclei were pelleted by low-speed centrifugation at 650 *g* for 3 min. Then the nuclei were resuspended in nuclear buffer containing 20 mM Tris–HCl (pH 7.5), 75 mM NaCl, 0.5 mM EGTA, 50% glycerol, 1 mM PMSF, 1 μl/ml RNase inhibitor, 1× phosphatase inhibitor and 1× protease inhibitor cocktail. They were incubated for 10 min at 4°C by the addition of 10 volumes of a buffer containing 20 mM HEPES (pH 7.6), 7.5 mM MgCl_2_, 0.2 mM EGTA, 300 mM NaCl, 1 M urea and 1% NP-40. The chromatin pellet was sedimented by centrifugation at 15 000 *g* for 10 min for caRNA isolation.

For RNA isolation, RNA was extracted from the chromatin pellet samples with TRIzol reagent according to the manufacturer's instructions. Briefly, these samples (500 μl) were mixed with 1 ml of TRIzol solution and extracted for 5 min by adding 200 μl of chloroform. Then, the upper aqueous phase containing RNA was separated from the samples by centrifugation (12 000 *g*, 10 min, 4°C) and transferred to a new 1.5 ml centrifuge tube for follow-up isopropanol precipitation and RNA isolation. Finally, isolated RNA was pre-treated with DNase I (9003–98-9, Merck), and 5 ng of RNA was reverse-transcribed to synthesize template DNA. qPCR was conducted with primers complementary to human or mouse snRNAs or the chromatin-associated HotAir ncRNA (control for data normalization) with TB Green Premix Ex Taq™ on the CFX Connect Real-Time System. The absence of contaminating genomic DNA was validated by the lack of amplified products for all sample/primer sets by the inclusion of control reverse transcription reactions in which enzyme was not added.

### Lentivirus-mediated targeted gene short hairpin RNA (shRNA) knockdown and overexpression

Lentiviral particles were generated by the transient transfection of HEK-293T cells following a standard protocol as described previously (36). Briefly, the *MEN1* knockdown, full-length *MEN1* and *RNase H1* sequence were constructed by inserting the corresponding sh*MEN1*, *MEN1* and *RNase H1* sequences into the pLVX-CMV-ZsGreen-Puro vector and pCDH-CMV-MCS-EF1-Puro vector, respectively. Subsequently, Chemi-Trans™ FectinBor DNA Transfection Reagent (T008, GeneCodex) was used to transfect the sh*MEN1*, *MEN1* and *RNase H1* overexpression, lentiviral skeleton and helper plasmids (pMD2.G, psPAX2) into HEK-293T cells. Lentiviral supernatant was harvested 72 h after transfection, and concentrated lentiviral medium containing 2 mg/ml polybrene was used to culture A549 cells or NCI-H446 cells. Infected cells were selected with 1 μg/ml puromycin for 2 weeks to obtain stable cell lines. The same cell lines were infected with a lentiviral vector with the green fluorescent protein (GFP) gene as a control. The shRNA sequence specifically targeting *MEN1* is 5′-GGAACCTGGCAGATCTAGA-3′.

### CRISPR/Cas9-mediated *MEN1* gene knockout

*MEN1*-KO NCI-H460 cell lines were established using the CRISPR/Cas9 [clustered regularly interspaced palindromic repeats (CRISPR)/CRISPR-associated protein 9] system as previously described (37). Briefly, a single-guide RNA (sgRNA) sequence targeting exon 3 of the *MEN1* locus (sg*MEN1* sequence: 5'-CAAATTGGACAGCTCCGGTGTGG-3') was designed on the basis of http://crispr.mit.edu, synthesized from Sangon Biotech and annealed. Subsequently, sg*MEN1* was digested using BsmBI (ER0452, Thermo Fisher Scientific) and ligated to the pX458 plasmid using a normal ligation reaction as recommended by the manufacturer (2011A, TaKaRa). The pX458-sg*MEN1* plasmid was transfected into NCI-H460 cells using electrotransfection. After 48 h of transfection, the cells were diluted by stepwise dilution to obtain single colonies. A GFP-positive cell cluster was selected to conduct genomic DNA sequencing, and cells displaying a single sequencing peak with a gap were considered candidate knockout cells.

### Isolation of chromatin-associated proteins

After three washes in ice-cold PBS, cells (1 × 10^7^) were resuspended in 200 μl of Buffer A containing 10 mM HEPES (pH 7.9), 10 mM KCl, 1.5 mM MgCl_2_, 0.34 M sucrose, 10% glycerol, 1 mM dithiothreitol (DTT), 10 mM NaF, 1 mM Na_2_VO_3_ and 1× protease inhibitor cocktail. They were incubated for 10 min at 4°C by the addition of Triton X-100 to a final concentration of 0.1%, followed by centrifugation at 1500 *g* for 5 min to separate the cytoplasmic proteins from the nuclei. Then, the extracted nuclei were lysed in 200 μl of Buffer B containing 3 mM EDTA, 0.2 mM EGTA, 1 mM DTT and protease inhibitor cocktail. Insoluble chromatin was gathered by centrifugation at 2000 *g* for 5 min, rinsed once with Buffer B and centrifuged at 12 000 *g* for 5 min. For the release of chromatin-associated proteins by MNase treatment, cell nuclei were resuspended in solution A and incubated with 0.25 U of MNase for 5 min at 37°C. The nuclease reaction was stopped by the addition of 1 mM EGTA and 1 mM EDTA. Nuclei were collected by centrifugation at 1200 *g* for 5 min, and protein abundance was detected by immunoblotting.

### Isolation of native non-cross-linked chromatin

Non-cross-linked chromatin was prepared as previously described (38), except MNase pre-treatment was substituted by a combination of DNase I digestion as recommended by the manufacturer and sonication for five cycles of 10 s on a JY92-IIN Ultrasonc Homogenizer (SCIETZ).

### Cell proliferation assay

The indicated cells were seeded onto 96-well plates at a density of 1500 cells per well. After 24 h of incubation, the cells were treated with various doses of Cisplatin (15663–271, Solarbio), etoposide (ETO) (IE0270, Solarbio), *N*-methyl-*N*'-nitro-*N*-nitrosoguanidine (MNNG) (924–16-3, Sigma), mitomycin C (MMC) (GC12353, GLPBIO), Madrasin (Mad) (HY-100236, MCE) or isoginkgetin (Iso) (HY-N2117, MCE). Cell viability was assessed by a Cell Counting Kit (CCK)-8 at the indicated time points according to the manufacturer's protocol (BS350, Biosharp).

### Colony formation assay

The indicated cells were seeded onto 6-well plates (1.5 × 10^3^ cells per well) for 24 h and treated with optimized concentrations of Cisplatin, ETO, MMC, Mad or Iso. The cells were cultured for ∼9 days and then stained with 0.5% crystal violet in methanol solution to determine colony formation efficiency. The cluster of stained cells was considered a colony at >50 cells.

### EdU incorporation and detection

A Click™ EdU-488 Cell Proliferation Detection Kit for Imaging (C0071S, Beyotime) was used to assay cell proliferation through EdU (5-ethynyl-2′-deoxyuridine) incorporation. Briefly, cells were seeded onto 96-well plates (1.0 × 10^3^ cells per well) and treated with dimethylsulfoxide (DMSO), Mad or Iso for 48 h. The cells were incubated with 10 μM EdU for 30 min. Then, samples were fixed and permeabilized, and the Click™ reaction was performed according to the manufacturer's instructions. Nuclei were stained with Hoechst 33342 for 15 min at room temperature in the dark. Plates were imaged with ImageXpress Micro-4 (Molecular Devices) or Nikon Ar1 (Nikon) microscopes. MetaMorph was used for image analysis. Data from nine different fields in each condition were quantified per experiment. The EdU entire population nuclear intensity and the percentage of cells incorporating EdU were measured. EdU intensity was also detected only for those cells that incorporated EdU.

### Flow cytometry for apoptosis detection

Cell apoptosis was determined by using an Annexin V-FITC/PI Apoptosis Detection Kit according to the manufacturer's protocol (40302ES60, Yeasen). Briefly, Mad-, Iso- and MNNG-treated cells were washed with ice-cold PBS and harvested by centrifugation at 1000 *g* for 5 min. Cells were resuspended in 100 μl of Binding Buffer and stained by adding 5 μl of Annexin V-FITC and 10 μl of propidium iodide (PI) Staining Solution for 15 min at room temperature in the dark before cell apoptosis analysis by flow cytometry (Cyto FLEX S, Beckman).

### Immunoblotting

The protein concentration was measured after the indicated cells were lysed with RIPA lysis buffer (P0013B, Beyotime), and 30 μg of total protein was separated by SDS–polyacrylamide gel electrophoresis (SDS−PAGE) and transferred to polyvinylidene difluoride (PVDF) membranes, and then subjected to immunoblotting to detect the expression of the indicated proteins following the standard procedure. The following primary antibodies were adopted: menin (A300-105A, Bethyl Laboratories, 1:1000), MLL1 (sc-374392, Santa Cruz, 1:1000), LEDGF (A300-847A, Bethyl Laboratories, 1:4000), SNRPA (10212–1-AP, Proteintech, 1:1000), U2AF65 (sc-53942, Santa Cruz, 1:1000), SF3B2 (10919–1-AP, Proteintech, 1:2000), histone H3 (4620, CST, 1:20 000), PRPF4 (10728–1-AP, Proteintech, 1:2000), SNRNP200 (23875–1-AP, Proteintech, 1:1000), SRSF2 (3195, CST, 1:1000), Pol II (ab264350, Abcam, 1:1000), p-Pol II (Ser2) (ab5095, Abcam, 1:1000), p-Pol II (Ser5) (2629, CST, 1:1000), DDX17 (ab24601, Abcam, 1:2000), RNase H1 (15606–1-AP, Proteintech, 1:1000), MBNL1 (66837–1-1g, Proteintech, 1:4000), phosoho-ATM (Thr1981) (ab81292, Abcam, 1:1000), γH2AX (80312, CST, 1:1000), GPX4 (67763–1-Ig, Proteintech, 1:1000), COX2 (27308–1-AP, Proteintech, 1:1000), Bcl-2 (68103–1-Ig, Proteintech, 1:1000) and cleaved caspase-3 (ab13847, Abcam, 1:1000). Next, after three washes in Tris-buffered saline–Tween (TBST) buffer, mouse or rabbit secondary antibodies were incubated with a 1:10000 dilution in 5% milk/TBST buffer for 1 h at room temperature. After more washes in TBST buffer, protein bands were visualized using Smart chemiluminescence (H31500-1, Lifesciences) on a Tanon Imaging System (Tanon-5200, Tanon Science Technology) and densitometry was performed by ImageJ software.

### S9.6 dot blot

Genomic DNA was extracted with a TIANamp Genomic DNA Kit (DP304-03, Tiangen), except that samples were treated without RNase A according to the manufacturer's instructions. After quantification by a UV/VIS spectrophotometer (UV5Nano, Mettler Toledo), 1 mg of DNA from each sample was blotted on a nylon membrane (Amersham) with a dot blot apparatus and vacuum suction. For RNase H treatment, 1 mg of DNA was incubated with RNase H at 37°C overnight, then extracted with a TIANamp Genomic DNA Kit. The membrane was denatured for 10 min in a solution containing 0.5 M NaOH and 1.5 M NaCl, and neutralized in a solution containing 1 M NaCl and 0.5 M Tris–HCl pH 7.0 for another 10 min. Before being completely dried, the membranes were cross-linked with UV (1200 mJ/cm^2^) and stained in 1% methylene blue solution (G1303, Solarbio) for 10 min. Subsequently, the membranes were washed with TBST buffer, blocked in 5% (w/v) milk in TBST buffer for 1 h at room temperature and incubated with S9.6 antibody (1:500) overnight at 4°C. After several washes in TBST buffer, the blots were incubated with IgG secondary antibody (1:1000) in TBST buffer for 1 h at room temperature. The blots were visualized using chemiluminescence on a Tanon Imaging System and quantified by ImageJ software.

### Immunohistochemistry (IHC)

Human lung cancer tissue or nude mouse tumor tissue was fixed in 4% paraformaldehyde solution, embedded in paraffin and sectioned (5 μm) onto glass slides. IHC staining was conducted as described previously (21) using primary antibodies against menin (A300-105A, Bethyl Laboratories, 1:4000), γH2AX (80312, CST, 1:1000) and Ki67 (9129, CST, 1:400). Negative controls were treated identically except no primary antibody was added. Pictures were captured with an Olympus VS200 SLIDEVIEW microscope with panoramic scan. For the quantification of IHC staining, six immunostained images with a final ×400 magnitude were randomly obtained for each section sample. ImageJ Pro Plus was used to calculate the level of immunostaining. The images were converted into 8-bit grayscale. Image regions were selected for the measurement of area and integrated density, and background intensity was measured by selecting three distinct areas in the background with no staining. The corrected optical density (COD) was determined as follows: COD = ID – (A × MGV), where ID is the integrated density of the selected image region, A is the area of the selected image region and MGV is the mean gray value of the background readings.

### Immunofluorescence (IF) assay

Cells were seeded onto polylysine-coated coverslips for 24 h and fixed in 4% paraformaldehyde for 10 min at room temperature. After three washes with PBS, the coverslips were permeabilized in PBS containing 0.1% Triton X-100 for 10 min and rinsed three times in PBS. Coverslips were blocked in PBS containing 5% bovine serum albumin (BSA) for 2 h at room temperature and then incubated with primary antibodies overnight at 4°C in a humid chamber. The following antibodies were adopted: γH2AX (80312, CST, 1:200), 53BP1 (NB-100–305, Novus, 1:100), S9.6 (MABE1095, Merck Milipore, 1:200), RNase H1 (15606–1-AP, Proteintech, 1:200) and menin (A300-105A, Bethyl Laboratories, 1:200). After three washes in PBS, secondary antibodies were incubated with a 1:200 dilution in 1% BSA for 1 h at 37°C in the dark. Coverslips were rinsed three times and stained with Hoechst (B2261, Sigma) for 15 min at room temperature in the dark. After more times washes in PBS, coverslips were mounted on microslides. Images were captured with a confocal microscope (Olympus SpinSR10).

### Co-immunoprecipitation (co-IP) assay

Plasmids WT Menin-Flag and mutant (Mut) Menin-Flag were subcloned into the lentivirus plasmids. The substitution of amino acid residue M278W in menin was performed using a site-directed mutagenesis kit (200518, Stratagene) according to the manufacturer's protocol. After more washes with ice-cold PBS, cells were lysed for 30 min at 4°C in NP-40 lysis buffer (ST2045, Beyotime) containing 1 μg/ml PMSF, phosphatase inhibitor and 1× protease inhibitor cocktail. The lysates were collected by centrifugation (12 000 *g*, at 4°C). After quantification of protein concentrations, 1 mg of protein was loaded onto pre-washed protein A/G magnetic beads for 30 min at 4°C. Then, the lysates were immunoprecipitated with the indicated antibodies overnight at 4°C. Next, the protein A/G magnetic beads were added and incubated for 2 h at 4°C to recover the IP complexes. The beads were washed five times with NP-40 buffer using a magnetic separator. The bound proteins were eluted with 2× SDS buffer and subjected to immunoblotting.

### Neutral comet assay of DNA double-strand breaks

We performed the neutral comet assay using a Trevigen CometAssay® Kit as recommended by the manufacturer with 1× TBE running buffer. Briefly, ∼2 × 10^5^ treated cells were resuspended in 0.1% low-melting point agarose in PBS. A 50 μl aliquot of the cell suspension was pipetted onto the indicated regions of CometSlides® and allowed to solidify for 15 min at 4°C. The following operations were performed under dark conditions. Cells were lysed for 1 h at 4°C in CometAssay Lysis Solution and then immersed in neutral electrophoresis buffer (NEB; Tris-acetate pH 9.0) for 30 min at 4°C. Slides were transferred to an electrophoresis tank filled with NEB and electrophoresed with a constant current of 1.0 V/cm for 45 min at 4°C. The slides were removed from the electrophoresis tank and placed flat in DNA precipitation buffer (1 M NH_4_Ac in 95% ethanol) for 30 min at room temperature. Subsequently, the slides were transferred into 70% ethanol for an additional 30 min and then dried overnight at room temperature. Next, sample DNA was stained with 1× EB diluted 1:10 000 in Tris-EDTA buffer pH 7.5. Comet images were captured at ×10 magnification with an IX71 epifluorescence microscope (Olympus, Japan). The percentage tail moments of >150 nuclei per sample were measured with OpenComet Assay software (39). In box and whisker plots, box and whiskers indicate the 25th–75th and 10th–90th percentiles, respectively, with lines representing median values.

### Alternative splicing assay

*MEN1*-WT and *MEN1*-KO NCI-H460 cells were transfected with the pMTE1A plasmid using Chemi-Trans™ FectinBor DNA Transfection Reagent following the manufacturer's protocol. After 48 h of transfection, total RNA was extracted using TRIzol reagent. pMTE1A AS was analyzed by RT–PCR with 0.5 μg of RNA using the high-capacity cDNA reverse transcription Kit (11141ES60, Yeasen). PCR was carried out with the exon 1 forward primer (5'-GTTTTCTCCTCCGAGCCGCTCCGA-3') and the exon 2 reverse primer (5'-CTCAGGCTCAGGTTCAGACACAGG-3') by using the following program: 94°C for 5 min, 32 cycles of 94°C for 45 s, 62°C for 30 s, 72°C for 1 min and a final step of 72°C for 10 min. PCR products were run in 2% agarose gels stained with 0.1‰ EB dye. Bands were visualized using a GleUV system. Band intensities were quantified using ImageJ software.

### Splice site motif analysis

Splice site motif scores were calculated using the matrices for splice sites available in ESEfinder version 3.0 (http://rulai.cshl.edu/tools/ESE/). The matrices for splice sites were derived from constitutive exons, and the thresholds correspond to the first quantile of all splice site scores.

### Luciferase/β-gal double-reporter assay

Cells were seeded onto 12-well plates (2 × 10^5^ cells per well) and transfected with the pTN24 splicing reporter plasmid with a constitutively expressed β-galactosidase (β-gal) reporter for transfection normalization and a luciferase (Luc) reporter that was conditional on the excision of a translational stop codon by splicing. The cells were collected 48 h after transfection, and the reporter activity was measured with a Dual-Light Reporter Assay System (Applied Biosystems) and determined by calculating the ratio of Luc to β-gal activity.

### Inhibition and re-initiation of transcription

Cells were cultured in 60 mm dishes to 60–70% confluency and then treated with 100 μM 5,6-dichlorobenzimidazole 1-β–d-ribofuranoside (DRB) (C4798, APExBIO) in complete medium for 5 h. The cells were washed three times with PBS to remove the DRB and cultured again in fresh complete medium for different amounts of time. The treated cells were harvested at 5 min intervals after the removal of DRB, and total RNA was extracted with TRIzol reagent according to the manufacturer's instructions. The expression of utrophin pre-mRNA was detected by qPCR using the primers spanning exon–intron junctions. The elongation rate of Pol II transcription was determined as described previously (40).

### Human lung cancer specimens

All specimens used in this study were obtained with informed consent according to protocols approved by the Human Ethics Committee of Guizhou Medical University (no. 2021-53). Human research procedures were performed in strict accordance with the Declaration of Helsinki. We collected 52 lung cancer samples: 42 lung adenocarcinoma (LUAD) samples, 8 squamous carcinoma samples, 1 small cell lung cancer (SCLC) sample and 1 large cell neuroendocrine carcinoma sample. We also collected corresponding adjacent non-cancerous specimens (>2 cm from the cancerous tissue) from patients who had undergone resection. The patients were diagnosed with LUAD, squamous carcinoma and SCLC by pathologists at the Affiliated Hospital of Guizhou Medical University.

### LUAD patient survival analysis

mRNA splicing pattern data and clinical parameters of LUAD cohorts were downloaded from the TCGA database portal (http://bioinformatics.mdanderson.org/TCGASpliceSeq and https://tcga-data.nci.nih.gov/tcga/, respectively). A total of 502 LUAD patients with fully characterized tumors were included in this study. To determine the correlation between levels of gene splicing isoforms and LUAD patients’ overall survival (OS), we divided the LUAD patients into high-PSI and low-PSI groups by the median cut-off and then conducted Kaplan–Meier survival analysis. We determined the correlation between gene expression and LUAD patient survival using a microarray RNA-seq dataset from a LUAD cohort (*n* = 502) by setting the online (http://ualcan.path.uab.edu/) Kaplan–Meier plotter tool for the optimal cut-off for the automatic separation of patients into high- and low-gene expression groups.

### Xenograft experiment *in vivo*

*MEN1*-WT or *MEN1*-KO NCI-H460 cells (4 × 10^6^ cells per mouse) were inoculated subcutaneously into the axillae of 6-week-old male BALB/C nude mice (Beijing HFK Bioscience Co., Ltd, Beijing, China), and tumor volumes were monitored every day with a caliper. Tumor volumes were calculated with the modified ellipsoidal formula as 1/2 × (longest diameter) × (shortest diameter)^2^. Mad (4 mg/kg) in 90% corn oil containing 10% DMSO was injected i.p. once daily for 12 days after the tumors had grown to ∼200 mm^3^. The mice were humanely killed after 2 weeks of Mad treatment. The xenograft tumors were dissected, and their weights were measured.

### Statistical analysis and reproducibility

Data were analyzed using the two-tailed Student's *t*-test for pairwise comparison, one-way analysis of variance (ANOVA) for multiple comparisons or log-rank tests for Kaplan−Meier survival analysis. When data were expressed as scatter plots, the Mann–Whitney *U*-test was performed. Test details are indicated in the figure legends. Statistical analyses were performed using the GraphPad Prism 8 package. Data are represented as means ± standard deviation (SD) or standard error of the mean (SEM) of at least three independent experiments. *n* values indicate biologically independent samples and experiments, and a *P*-value <0.05 was considered statistically significant (with **P < 0.01, ***P < 0.001 and ****P < 0.0001 indicating higher levels of significance). Statistical parameters can be found in the figure legends.

## RESULTS

### *MEN1* deficiency disrupts global alternative pre-mRNA splicing profiles

**Figure 1.**
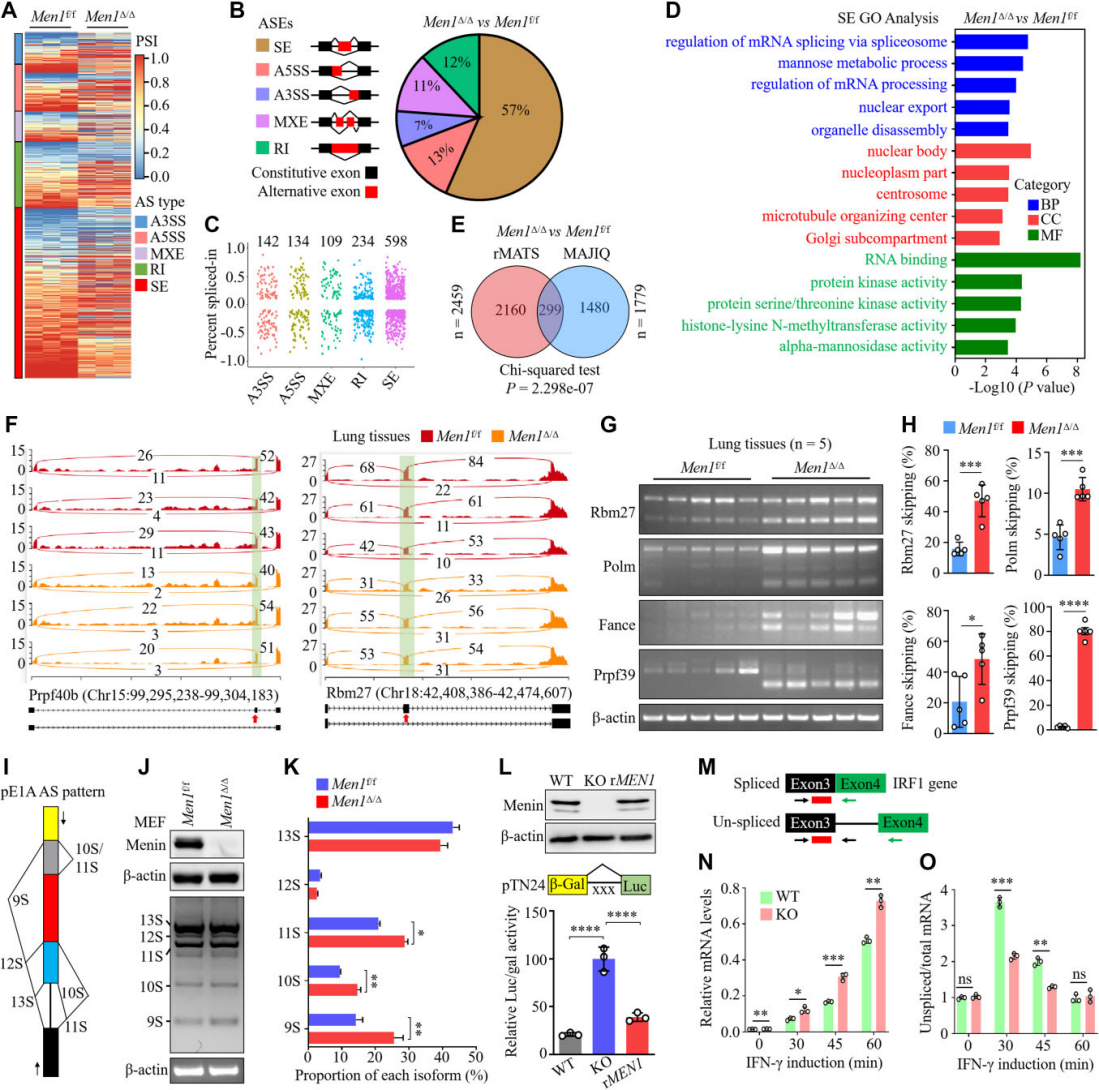
*MEN1* deficiency disrupts global alternative pre-mRNA splicing profiles. (**A**) Heatmap showing PSI values for differentially spliced ASEs between *Men1*^f/f^ and *Men1*^Δ/Δ^ mouse lung tissue. The PSI between *Men1*^Δ/Δ^ and *Men1*^f/f^ mouse lung tissue was based on three independent biological samples from each group. (**B**) Schematic (left) and distribution (right) of five AS types that differentially change upon *Men1* loss in mouse lung tissue. (**C**) Dot plot showing the distribution of PSI values for five AS types upon *Men1* loss in mouse lung tissue. (**D**) GO enrichment analysis showing biological processes (BPs), cellular components (CCs) and molecular functions (MFs) affected by *MEN1*-regulated SE events in mouse lung tissue. (**E**) Venn diagram showing the intersection of ASEs between the rMATS and MAJIQ datasets. The *P*-value was determined with the χ^2^ test. (**F**) Representative Sashimi plot showing the increased SEs of the *Prpf40b* and *Rbm27* genes in *Men1*^Δ/Δ^ mouse lung tissues compared with *Men1*^f/f^ mice. Green highlights indicate AS events, with the number of junction reads indicated for each event. (**G**) Validation by RT−PCR of the indicated genes with SE events identified by MAJIQ and rMATS in *Men1*^f/f^ and *Men1*^Δ/Δ^ mouse lung tissue (*n* = 5 mice per group). (**H**) Quantification of skipping rates of the indicated genes for the experiments in (G). Dots in the histogram depict individual samples. (**I**) Diagram of the E1A reporter gene indicating A5SSs and splicing events that generate 13S, 12S, 11S, 10S and 9S mRNAs. Positions of the exon primers used for RT−PCR are shown as arrows. (**J**) RT−PCR and gel electrophoresis analysis of pMTE1A containing the E1A reporter gene transfected into *Men1*^f/f^ and *Men1*^Δ/Δ^ MEFs. (**K**) Quantification of the percentage of each splicing isoform for the experiments in (J). (**L**) The pTN24 minigene reporter (middle), consisting of β-gal, an upstream intron that contains three translational stop codons (× × ×) and luciferase (Luc), was transfected into *MEN1*-WT, *MEN1*-KO and *rMEN1* NCI-H460 cells. Immunoblotting for menin protein (top) and quantification of the ratios of Luc activity relative to β-gal activity (bottom); r*MEN1*, re-expression of wild-type *MEN1* in *MEN1*-KO NCI-H460 cells. (**M**) Schematic representation of the primer pairs used to detect the unspliced pre-mRNA and total mRNA. Arrows indicate the primers used. (**N**) qPCR was used to detect the total mRNA produced from the IRF1 gene after the indicated time points following treatment with interferon-γ (IFN-γ). (**O**) qPCR was used to monitor the ratio of unspliced to total mRNA of the IRF1 gene after the indicated time points following treatment with IFN-γ. In (G), (J) and (L), the images are representative of three independent experiments. In (H), (K), (L), (N) and (O), data are represented as the mean ± SD (*n* = 3 biologically independent experiments), analyzed by two-tailed unpaired *t*-test; *P < 0.05; **P < 0.01; ***P < 0.001; ****P < 0.0001.

To determine whether *MEN1* plays a role in AS, we profiled the transcriptome in whole-lung tissue from *Men1*^f/f^ and *Men1*^Δ/Δ^ mice by deep RNA-seq and then performed PSI analyses of the AS using rMATS (29). Gene expression analysis led to the identification of 444 DEGs (*P* < 0.05 and fold change ≥ 1.5), of which 210 (47.3%) were up-regulated and 234 (52.7%) were down-regulated in the *Men1*^Δ/Δ^ mice compared with the *Men1*^f/f^ mice (Supplementary Figure S1A; Supplementary Data S1). It is intriguing that the loss of *Men1* resulted in notable changes in the AS profiles (Figure 1A). Through an rMATS analysis, we identified 2459 ASEs that belonged to 2135 genes in the lung tissues of *Men1*^Δ/Δ^ mice (FDR < 0.05 and |ΔPSI| ≥ 0.1) (Figure 1B, C; Supplementary Figure S1B; Supplementary Data S2). The largest proportion of ASEs were skipped exons (SEs; 57%), followed by alternative 5' splice sites (A5SSs; 13%), retained introns (RIs; 12%), mutually exclusive exons (MXEs; 11%) and alternative 3' splice sites (A3SSs; 7%) (Figure 1B, C). Moreover, we noted that of the 444 identified DEGs, only 37 genes carried ASEs (Supplementary Figure S1C), and no relationship was found between the expression of these 37 genes and their PSIs (Supplementary Figure S1D), which suggests that alterations in AS patterns in the lung tissue of the *Men1*^Δ/Δ^ mice were unlikely to have been caused by changes in transcript levels. These results demonstrate that the loss of *Men1* results in an aberrant AS profile in mouse lung tissue.

GO analysis revealed that *Men1*-regulated ASEs were involved in RNA metabolism (such as mRNA processing, RNA splicing and RNA binding), nuclear speckles and mRNA splice site selection. Importantly, the terms enriched with these ASE-carrying genes included regulation of mRNA splicing via spliceosome, U2-type pre-spliceosome, U1 snRNP and spliceosomal complex assembly (Figure 1D; Supplementary Figure S1E–G). To further probe global AS affected by *Men1* loss during the RNA metabolic process, we reanalyzed RNA-seq data using MAJIQ, another AS analysis tool, to calculate both typical binary ASEs and intricate local splicing variants (33). We identified *Men1*-regulated splicing alterations totaling 1779 ASEs in the lung tissue of the *Men1*^Δ/Δ^ mice (Figure 1E). Venn diagrams generated via rMATS and MAJIQ verified with high confidence that the differential splicing of 299 genes was regulated by *Men1* deletion (Figure 1E). We also confirmed the aberrant splicing of a few genes, as exemplified by *Prpf40b*, *Rbm27*, *Polm*, *Fance* and *Prpf39*, by visualization using Sashimi plots (Figure 1F; Supplementary Figure S1H). Some of the aberrantly spliced genes in the lung tissue of *Men1*^Δ/Δ^ mice were validated via RT–PCR assays (Figure 1G, H). Similar findings were observed in the *Men1*^f/f^ and *Men1*^Δ/Δ^ MEFs (Supplementary Figure S1I).

Next, we evaluated whether AS is impacted by *MEN1* expression. To this end, we transiently transfected the adenovirus E1A minigene reporter plasmid pMTE1A carrying multiple 5' splice sites into the *Men1*^f/f^ and *Men1*^Δ/Δ^ MEFs. This minigene reporter can produce five mRNAs, sizes 13S, 12S, 11S, 10S and 9S, by AS (Figure 1I) (41). RT–PCR displayed that in the *Men1*^Δ/Δ^ MEFs, AS of the E1A reporter was impaired, as indicated by an increase in the 11S, 10S and 9S isoform levels compared with those in the *Men1*^f/f^ MEFs (Figure 1J, K). Furthermore, we carried out another splicing assay with NCI-H460 cells following the transduction of a double-reporter plasmid pTN24 expressing β-gal, through which luciferase was expressed only when suitable splicing excised an upstream intron sequence with translational stop codons (Figure 1L) (41). *MEN1*-KO dramatically promoted the ratio of spliced pTN24, as indicated by increased Luc/β-gal activity, whereas the reconstituted expression of wild-type *MEN1* (r*MEN1*) in the *MEN1*-KO cells reversed the promotion of *MEN1*-KO on pTN24 splicing efficiency (Figure 1L).

Finally, we measured the pre-mRNA splicing of the rapidly inducible gene IRF1. Primer sets were designed to detect the intron-containing pre-mRNA (unspliced primer set) and the total mRNA (spliced primer set) that is produced upon IRF1 induction (Figure 1M). Transcripts of the IRF1 gene were detected when using the spliced primer set, and IRF1 mRNA levels were monitored 0, 30, 45 and 60 min after the addition of interferon-γ (IFN-γ) (Figure 1N). To determine pre-mRNA splicing, we considered the ratio of unspliced to total mRNA (splicing efficiency). Under normal conditions, the ratio of unspliced to total mRNA was highest 30 min after IRF1 induction, which indicates that splicing is a slow step relative to transcription and increases over time during the generation of mRNA. We further analyzed the relative splicing efficiency of IRF1 in the *MEN1*-KO cells compared with the *MEN1*-WT cells. It is striking that 30 min after the induction of IRF1, we observed a 1.8-fold decrease in the ratio of unspliced to total mRNA in the *MEN1*-KO cells compared with the *MEN1*-WT cells. The effect on splicing was diminished 45 min after induction of IRF1, and no effect was observed after 60 min of induction (Figure 1O). These results demonstrate that *MEN1* deficiency enhances the splicing efficiency of IRF1 pre-mRNA. Taken together, these findings clearly demonstrate that *MEN1* is a key regulator of alternative RNA splicing and that its deficiency disrupts the homeostasis of the global splicing network.

### *MEN1* alters exon skipping and the abundance of RNA splicing isoforms

The generalizability of the effect of *MEN1* on AS patterns was strongly corroborated by repeated RNA-seq and differential exon usage analyses with the *MEN1*-WT and *MEN1*-KO NCI-H460 cells (Supplementary Data S3 and S4). Venn diagram analysis identified 399 conserved *MEN1*-regulated ASEs from the mouse lung tissue and lung cancer cells, 164 of which were SE events (Supplementary Figure S2A). We further confirmed the differential splicing of several conserved genes, such as *SENP6*, *EMSY*, *YTHDF3* and *GGPS1*, by visualization using Sashimi plots (Supplementary Figure S2B). Because SE events represent the vast majority of *MEN1* deletion-related ASEs (57% in mouse lung tissue and 62.2% in NCI-H460 cells) (Figure 1B; Supplementary Data S4), we investigated the underlying mechanism by which menin regulates exon skipping.

**Figure 2.**
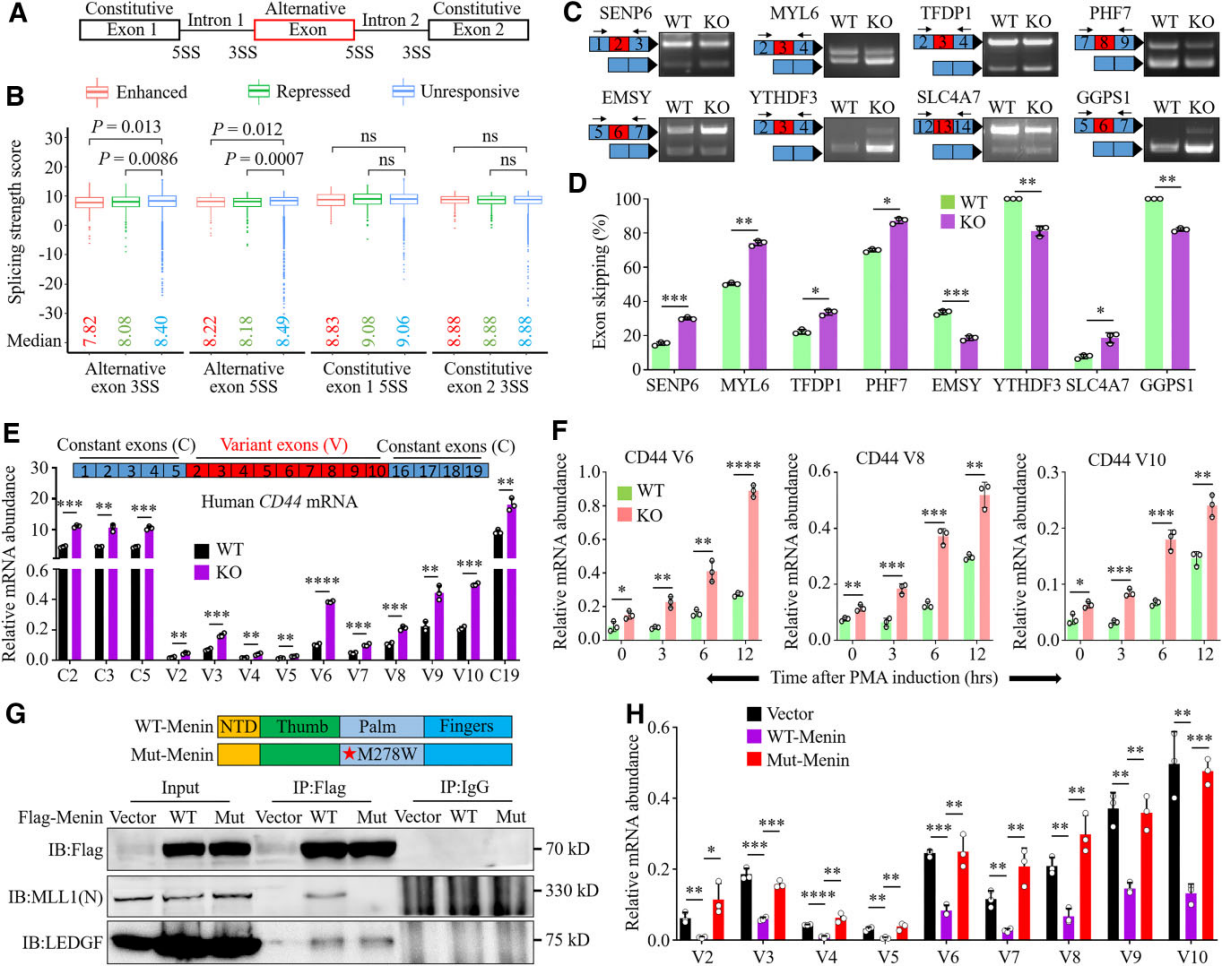
*MEN1* alters exon skipping and the abundance of RNA splicing isoforms. (**A**) Schematic of alternative and constitutive exons. 5' and 3' splicing sites (5SS and 3SS) are indicated accordingly. (**B**) Box plots of splice site scores calculated for *MEN1*-regulated SEs. Box plots show the median (the horizontal line in the box), 25th and 75th percentiles (lower and upper bounds of the box, respectively), and minimum and maximum (lower and upper whiskers, respectively). *P*-values were determined by two-sided unpaired Wilcoxon tests. (**C**) RT−PCR and gel electrophoresis analysis of the indicated SE genes in *MEN1*-WT and *MEN1*-KO NCI-H460 cells. Information for target exons is shown in the left panel. Blue box, constitutive exon; red boxes, skipped exon. (**D**) Quantification of skipping rates of the indicated SE genes for the experiments in (C). (**E** and **F**) qPCR was used to detect the mRNA abundance of the CD44 constant (C) and variant exons (V) in *MEN1*-WT and *MEN1*-KO NCI-H460 cells treated with or without 40 ng/ml PMA for the indicated times. (**G**) Diagram of WT-Menin and Mut-Menin M278W structure domains (top). *MEN1*-KO NCI-H460 cells were infected with the empty virus (vector), WT-Menin and Mut-Menin M278W lentivirus-expressing plasmids, and Co-IP was performed with 3× Flag-menin or IgG antibodies, followed by immunoblotting for menin, MLL1 and LEDGF proteins (bottom). (**H**) qPCR was used to detect the mRNA abundance of the indicated CD44 variant exons in *MEN1*-KO NCI-H460 cells transfected with vector, WT-Menin or Mut-Menin plasmids for 72 h. In (C) and (G), images are representative of three independent experiments. In (D), (E), (F) and (H), data are represented as the mean ± SD (*n* = 3 biologically independent experiments), analyzed by two-tailed unpaired *t-*test; *P < 0.05; **P < 0.01; ***P < 0.001; ****P < 0.0001.

By comparing splice site scores of responsive and unresponsive SE-forming events, we found that both *MEN1*-enhanced and *MEN1*-repressed exons had weaker 5' splice sites (5SSs) or 3SSs than those observed in unresponsive SEs (Figure 2A, B). We analyzed the features of the splice sites of *MEN1*-regulated and *MEN1*-independent genes using the matrices for splice sites available in ESEfinder (42). We were surprised to find that *MEN1*-regulated SE genes, but not all differentially spliced genes, harbored a weak 5SS or 3SS. Some of these genes were associated with motif scores that were below the threshold score for classic constitutive splice sites; more intriguing is that some splice site scores, such as those for the *TFDP1*, *ZNF18* and *SLTM* genes, were similar to those for standard constitutive splice sites but corresponded to bispecific splice sites (Supplementary Figure S2C, right), which may confuse the splicing components because they may be recognized as either 5SS or 3SS (43). We designed several PCR primer pairs targeting two neighboring constitutive exons in a cluster of *MEN1*-regulated genes with SEs. Knocking out *MEN1* in NCI-H460 cells resulted in a marked increase in *SENP6*, *MYL6, TFDP1*, *PHF7* and *SLC4A7* exon skipping, whereas it inhibited exon skipping in *EMSY*, *YTHDF3* and *GGPS1* genes (Figure 2C, D). In agreement with this finding, shRNA-mediated genetic knockdown of *MEN1* (sh*MEN1*) in A549 cells yielded similar results (Supplementary Figure S2C, left). In contrast, overexpression of *MEN1* reduced the isoform abundance of *SENP6*, *ZNF18*, *PHF7*, *CDH1* and *TFDP1* but promoted *SLTM* isoform generation in NCI-H446 cells (Supplementary Figure S2D, E). Our data suggest that the presence of suboptimal 5SSs or 3SSs may render splicing of these genes largely dependent on *MEN1* for their efficient recognition by spliceosomal proteins and subsequent AS.

The human CD44 gene consists of 10 constitutive exons (C1–C5 and C16–C19) and 9 clustered variable exons (V2–V10), and produces multiple splice variants, some of which are involved in the development and progression of various tumors (44). We found that knockout of *MEN1* promoted the generation of constant and variant CD44 exons (Figure 2E); treatment with the phorbol ester PMA (phorbol 12-myristate 13-acetate), an activator of the protein kinase C (PKC) pathway, enhanced the *MEN1* KO-induced accumulation of CD44 V6, V8 and V10, and did so in a time-dependent manner, as determined by qPCR using primers specific for constant or variable exons (Figure 2F). Alterations in the abundance of RNA splicing isoforms can be caused by changes in AS processes or differential isoform degradation rates. Here, our results showed that *MEN1* affects the AS process of CD44 genes, not their degradation, as inhibiting transcription with actinomycin D (Act.D) did not alter the effect of *MEN1* (Supplementary Figure S2F, G).

Next, we investigated whether *MEN1* regulates AS in a manner dependent on mixed lineage leukemia protein 1 (MLL1), a histone H3 lysine 4 methyltransferase that interacts with menin (45). qPCR results showed that treatment with MI-3, a specific inhibitor of the menin–MLL1 interaction (46), strikingly enhanced the abundance of C5, C19 and variable CD44 exons (V2–V10) in NCI-H446 cells treated with or without PMA (Supplementary Figure S2H, I). To consolidate this finding, we stably transfected plasmids expressing WT-Menin or mutant Menin Met278Trp (Mut-Menin) into *MEN1*-KO NCI-H460 cells. Consistent with the previous reports (45), Co-IP data indicated that a menin Met278Trp substitution completely disrupted the interaction of menin with MLL1 but not the menin–LEDGF (lens epithelium-derived growth factor) interaction (Figure 2G). As expected, ectopic expression of WT-Menin, but not of Mut-Menin, profoundly prevented the generation of variable CD44 exons (Figure 2H). These results suggest that the MLL1-dependent effects of *MEN1* contribute to its regulation of AS.

### *MEN1* regulates AS by slowing the Pol II elongation rate

**Figure 3.**
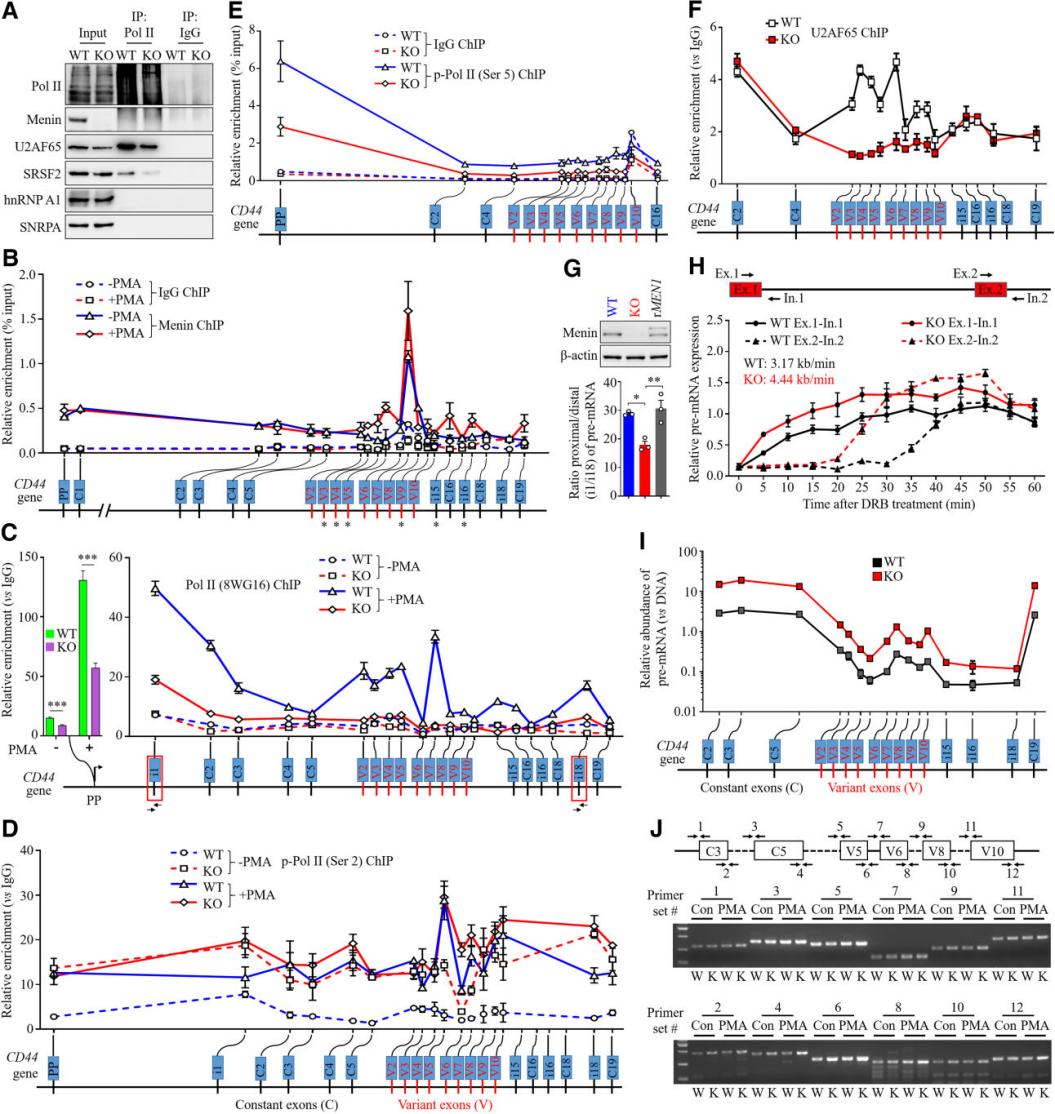
*MEN1* regulates AS by slowing the Pol II elongation rate. (**A**) Chromatin-associated proteins were extracted from *MEN1*-WT and *MEN1*-KO NCI-H460 cells, and IP was performed with Pol II or IgG antibodies, followed by immunoblotting for the indicated proteins. (**B–F**) Chromatin was extracted from *MEN1*-WT and *MEN1*-KO NCI-H460 cells treated with or without 40 ng/ml PMA for 6 h and ChIP walking assays were performed with antibodies against menin (B), Pol II (8WG16) (C), p-Pol II (Ser2) (D), p-Pol II (Ser5) (E) and U2AF65 (F), followed by qPCR to detect the enrichment of these proteins on the *CD44* gene locus. Amounts of menin and p-Pol II (Ser5) are expressed as percent input, and the amounts of Pol II (8WG16), p-Pol II (Ser2) and U2AF65 are expressed relative to the signal obtained for ChIP using IgG. (**G**) Quantification of ratios of pre-mRNA abundance at CD44 intron 1 (i1) to that at intron 18 (i18) in *MEN1*-WT, *MEN1*-KO and r*MEN1* NCI-H460 cells (bottom). Validation of menin expression by immunoblotting (top). (**H**) Top: schematic diagram showing the primer sets used to amplify the exon–intron junctions of exons 1 and 2 of the utrophin gene. Bottom: first *MEN1*-WT and *MEN1*-KO NCI-H460 cells were treated with 100 μM DRB for 3 h, and then fresh medium was added after DRB removal. The cells were collected at 5 min intervals, and qPCR was used to measure the expression of pre-mRNA in the exon 1 and exon 2 regions of the utrophin gene. Expression is plotted relative to the expression level of the no treatment control, which was set to 1 in all experiments. (**I**) qPCR quantification of CD44 pre-mRNA exons retained on template chromatin. Non-cross-linked DNase-treated chromatin from *MEN1*-WT and *MEN1*-KO NCI-H460 cells was extracted as described in the Materials and Methods. The graph displays the means ± SD of chromatin-associated pre-mRNA relative to the DNA in the input (quantified by qPCR). (**J**) Schematic diagram of constant exons (C3 and C5) and variant exons (V5, V6, V8 and V10) of the CD44 gene and the primer sets designed for RT−PCR shown (top). Chromatin-associated snRNAs were isolated, and RT−PCR analysis was performed with primer sets to compare the chromatin abundance of CD44 pre-mRNA in *MEN1*-WT (W) and *MEN1*-KO (K) NCI-H460 cells treated with or without PMA. In (A), (G) and (J), images are representative of three independent experiments. Data are represented as the mean ± SEM (B–F) or the mean ± SD (G–I) (*n* = 3 biologically independent experiments), analyzed by two-tailed unpaired *t*-test; *P < 0.05; **P < 0.01; ***P < 0.001; ns, not significant.

The aforementioned findings prompted us to continue to explore how *MEN1* regulates alternative RNA splicing. Because menin did not interact with the splicing factors evaluated, we ruled out the possibility that *MEN1*-affected AS is mediated through protein–protein interactions (Supplementary Figure S3A). We speculated that *MEN1* might impact the recruitment of splicing factors to Pol II, which in turn would change the AS process. Co-IP showed that Pol II was associated with splicing factors, including SRSF2 and U2AF65, but not with SNRPA or hnRNPA1 in the *MEN1*-WT cells (Figure 3A). It is important to note that the interaction of SRSF2 or U2AF65 with Pol II was greatly reduced in the *MEN1*-KO cells (Figure 3A). These results suggest that menin facilitates the recruitment of key spliceosomal factors, such as SRSF2 and U2AF65, to elongate Pol II complexes during transcription.

The slowing of the Pol II elongation rate prevented exon skipping by increasing the chances for recruitment and recognition of the splicing machinery to weak splice sites (47). The requirement for simultaneous transcription to induce an effect of *MEN1* on AS drew our attention to the abnormal AS patterns induced by *MEN1* deletion on Pol II elongation. ChIP walking assays with NCI-H460 cells showed that menin was present not only in the promoter regions of the CD44 gene but also in exon and intron regions; this protein appeared to be preferentially positioned at variant exons, particularly in V5, V6, V7 and V8, relative to constant exons or their neighboring regions (Figure 3B). PMA treatment significantly increased the abundance of menin inside the CD44 V3, V4, V5, V9, i15 and i16 regions (Figure 3B). Similarly, widespread distribution of Pol II, Ser5-phosphorylated Pol II [p-Pol II (Ser5)], a mark of paused Pol II (48), and p-Pol II (Ser2) on the CD44 gene was also observed, and PMA stimulation resulted in the accumulation of these Pol II proteins within either constant exons or variant exons (Figure 3C–E). Importantly, knocking out *MEN1* dramatically decreased the chromatin enrichment of Pol II and p-Pol II (Ser5) on the CD44 gene and enhanced the accumulation of p-Pol II (Ser2) (Figure 3C–E), but exerted no affect the chromatin modifications of histone H3 (Supplementary Figure S3B). The accumulation of p-Pol II (Ser2) induced by *MEN1* deletion was not further augmented by PMA, presumably because 100% abundance was reached at these regions (Figure 3D). Immunoblotting showed that *MEN1*-KO notably decreased the enrichment of chromatin with Pol II, p-Pol II (Ser5) and total Rbp1 C-terminal domain (CTD; phosphorylated and unphosphorylated Pol II forms), and increased p-Pol II (Ser2) accumulation, although treatment with Cisplatin reduced the chromatin association of these Pol II proteins in a time-dependent manner (Supplementary Figure S3C). Importantly, the deletion of *MEN1* reduced recruitment of U2AF65 to the variant exons but not to the constant exon and intron regions (Figure 3F), and moderately increased the recruitment of the U1 snRNP protein SNRPA to the CD44 gene regions (Supplementary Figure S3D). These findings suggest that *MEN1* regulates AS by slowing the Pol II elongation rate and by facilitating recruitment of splicing machinery either directly or through Pol II.

An independent evaluation of the impacts of *MEN1* on the elongation rate of RNA polymerase involved analyzing the distribution of Pol II and p-Pol II (Ser2) along the *c-Myc* gene (49). Protein occupancy was detected at 17 positions within the *c-Myc* gene and its flanking sequences in the *MEN1*-WT and *MEN1*-KO NCI-H460 cells. We observed that Pol II occupancy was elevated at the transcription start site and decreased in other gene regions (Supplementary Figure S3E), whereas the high occupancy of p-Pol II (Ser2) in the *c-Myc* gene was noted in a region from +4828 to +7028 bp of the poly(A) site (Supplementary Figure S3F). Notably, *MEN1* deletion markedly reduced the occupancy of Pol II throughout the gene while increasing the enrichment of p-Pol II (Ser2) (Supplementary Figure S3E, F). To corroborate the evidence for the regulation of Pol II elongation by *MEN1*, we made use of an observation by others suggesting that transcription mediated by a slow mutant human Pol II led to an increased promoter-proximal to promoter-distal pre-mRNA ratio (47). We isolated pre-mRNAs from the nuclei of *MEN1*-WT and *MEN1*-KO cells and performed qPCR with primer sets located at each end of the CD44 gene (intron 1 and intron 18). Knocking out *MEN1* resulted in a 1.5-fold decrease in the ratio of intron 1 to intron 18, which was reversed by re-expression of *MEN1* (r*MEN1*) (Figure 3G).

We validated this fast Pol II progression induced by *MEN1* deletion by using the DRB release method to calculate the rate of *in situ* Pol II elongation on the utrophin gene. Transcription of the exon 1 region of the utrophin gene was able to recover within minutes of DRB release in both cell types, and pre-mRNA expression in *MEN1*-KO cells was higher than that in *MEN1*-WT cells (Figure 3H). Reciprocally, in *MEN1*-WT NCI-H460 cells, the recovery of expression of the exon 2 region was delayed until 35 min after drug removal, which is consistent with previous data (40); however, the delay in transcription of this gene was ∼20 min in *MEN1*-KO cells (Figure 3H). This is consistent with a transcriptional lag due to the genomic distance between the first two exons (the utrophin gene consists of 74 exons and 73 introns, and the first intron is 110 kb long). These data suggest that in *MEN1*-WT NCI-H460 cells, Pol II transcribed the 110 kb region of the utrophin gene in ∼30 min at a rate of 3.2 kb/min, whereas in the absence of *MEN1*, Pol II transcribed the same region of the utrophin gene within 20 min at a rate of 4.4 kb/min (Figure 3H).

A deficiency of *MEN1* expedited the processivity rate of RNA polymerase, which compelled us to examine the retention of nascent pre-mRNA on template chromatin. We extracted native non-cross-linked chromatin and sonicated it at a low frequency to obtain a fragment of relatively large RNA with transcriptionally active chromatin (38). qPCR was used to quantify pre-mRNA levels at different positions throughout the CD44 gene. We found that corresponding exons or introns encoded greater amounts of CD44 pre-mRNA in the *MEN1*-KO cells than in the *MEN1*-WT cells (Figure 3I). This finding was congruent with the results obtained after the depletion of Pol II and p-Pol II (Ser5) and the accumulation of p-Pol II (Ser2) observed in these gene regions (Figure 3C–E); all these results indicate that *MEN1*-KO accelerates the progression of Pol II elongation, which results in the retention of nascent unspliced transcripts on the DNA template. Similar to *MEN1*-regulated SE genes, most exons throughout the entire CD44 gene possess a weak 5SS or 3SS (Supplementary Figure S3G), which renders these exons increasingly prone to skipping and mis-splicing when *MEN1* is dysfunctional. Indeed, we observed that *MEN1* knockdown induced the accumulation of pre-mRNA splicing isoforms encoded by corresponding constant and variant exons (Supplementary Figure S3H) and that these effects were PMA inducible and time dependent (Supplementary Figure S3I, J). Furthermore, we designed PCR primers targeting certain exon–intron (5SS) or intron–exon (3SS) junctions within the CD44 gene, confirming that *MEN1* deletion resulted in the marked accumulation of pre-mRNA splicing isoforms at most splice sites (Figure 3J). Taken together, these experiments demonstrate that *MEN1* decreases the rate of RNA polymerase elongation and favors the use of the weak splice sites present in the *CD44* gene, thereby inducing the generation of variant exons or splicing isoforms.

### *MEN1* prevents R-loop-induced accumulation of DNA damage and genome instability

**Figure 4.**
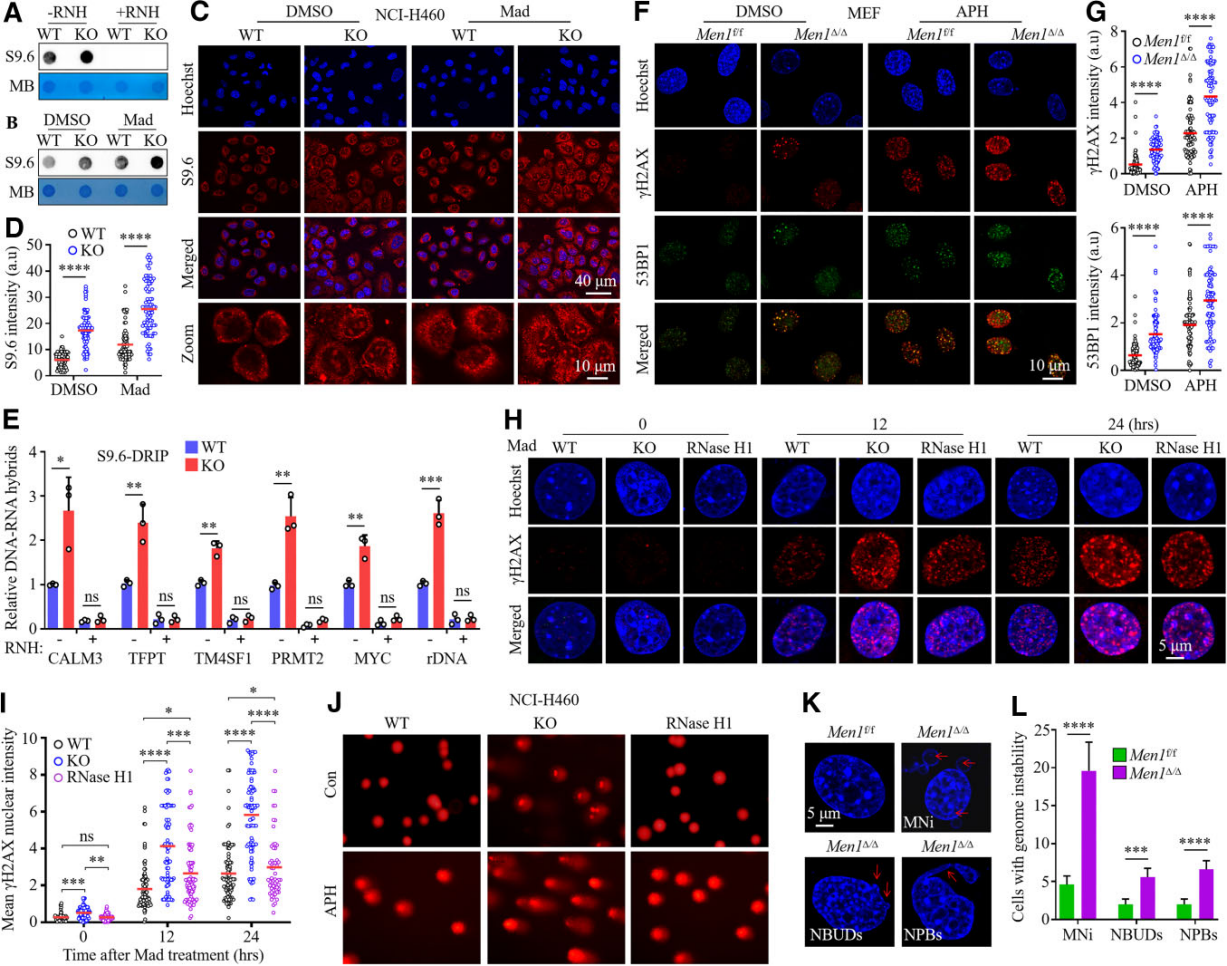
*MEN1* prevents R-loop-induced accumulation of DNA damage and genome instability. (**A** and **B**) Dot blot was used to detect global DNA−RNA hybrids with the S9.6 antibody in *MEN1*-WT and *MEN1*-KO NCI-H460 cells treated with or without 4 μM Mad for 24 h. A 1 mg aliquot of genomic DNA pre-treated with or without RNase H (RNH) was used to determine the spotted DNA content. MB, methylene blue. (**C** and **D**) IF staining (C) and intensity quantification (D) of S9.6 in *MEN1*-WT and *MEN1*-KO NCI-H460 cells treated with 4 μM Mad for 24 h; scale bars, 40 μm or 10 μm (zoom). Dots in the histogram in (D) depict individual cells, and >100 cells per condition were considered in each experiment. Median values are indicated by red lines (two-tailed Mann–Whitney *U*-test; ****P < 0.0001); a.u, arbitrary units. (**E**) DRIP-qPCR analysis of the *CALM3*, *TFPT*, *TM4SF1*, *PRMT2*, *MYC* and *rDNA* genes in *MEN1*-WT and *MEN1*-KO NCI-H460 cells. R-loop dissolution by RNH is shown as a negative control. Data are represented as the mean ± SD from three independent experiments (two-tailed paired *t*-test; *P < 0.05; **P < 0.01; ***P < 0.001). (**F** and **G**) IF staining (F) and intensity quantification (G) of γH2AX (red) and 53BP1 (green) in *Men1*^f/f^ and *Men1*^Δ/Δ^ MEFs treated with 0.4 μM aphidicolin (APH) for 24 h; scale bar, 10 μm. Dots in the histogram in (G) depict individual cells, and >100 cells per condition were considered in each experiment. Median values are indicated by red lines (two-tailed Mann–Whitney *U*-test; ****P < 0.0001). (**H**) *MEN1*-WT cells, *MEN1*-KO cells and RNase H1-overexpressing NCI-H460 cells with *MEN1* deletion were treated with 4 μM Mad, followed by IF staining for γH2AX (red) at the indicated time points; scale bar, 5 μm. (**I**) Quantification of γH2AX intensity for the experiments in (H). Dots in the histogram depict individual cells, and >100 cells per condition were considered in each experiment. Median values are indicated by red lines (two-tailed Mann–Whitney *U*-test; *P < 0.05; **P < 0.01; ***P < 0.001; ****P < 0.0001; ns, not significant). **(J**) Neutral comet assay in *MEN1*-WT, *MEN1*-KO and RNase H1-overexpressing NCI-H460 cells treated with 0.4 μM APH for 24 h. (**K** and **L**) Representative images of Hoechst staining showing MNi, NBUDs and NPBs in *Men1*^f/f^ and *Men1*^Δ/Δ^ MEFs treated with 4 μM Mad for 24 h (K). The statistical results were obtained from an analysis of mitotic cells (*n* = 100 per group) (L); the count was repeated three times and data are represented as the mean ± SD, analyzed by two-tailed unpaired *t*-test; ***P < 0.001; ****P < 0.0001; scale bar, 5 μm.

Disrupting the recruitment of splicing factors to Pol II prolongs the association of naked nascent RNA with single-stranded template DNA, thereby resulting in three-stranded nucleic acid structures known as R-loops (50). Unscheduled R-loops lead to DNA damage and genome instability after the depletion of splicing factors, which in turn contribute to cancer (51–53). In this study, we first monitored genomic DNA extracted from *MEN1*-WT and *MEN1*-KO NCI-H460 cells with the monoclonal antibody S9.6, which specifically detects DNA−RNA hybrids (54). We observed a significant increase in the enrichment of DNA−RNA hybrids in the *MEN1*-KO cells that was abolished by treating the DNA with RNase H, which binds and hydrolyzes the RNA strand of DNA−RNA duplexes (Figure 4A; Supplementary Figure S4A) (55), validating the specificity of the S9.6 antibody. It is interesting that treating NCI-H460 cells with Mad, a splicing inhibitor that disrupts early-stage spliceosome assembly and arrests at spliceosome complex A (56), also resulted in abundant R-loop formation, which was enhanced by the deletion of *MEN1* (Figure 4B; Supplementary Figure S4B). IF staining showed that the S9.6 signal was present in both the cytoplasm and nucleus; the *MEN1*-deleted cells were significantly enriched in DNA−RNA hybrids, in particular in the nucleus, relative to the *MEN1*-WT cells (Figure 4C, D). The deletion of *MEN1* promoted Mad-induced R-loop formation (Figure 4C, D), which was consistent with the S9.6 dot blot results. Similar findings were revealed for *Men1*^f/f^ and *Men1*^Δ/Δ^ MEFs, which showed that in the absence of *MEN1*, MEFs exhibited conspicuous accumulation of nucleolar DNA−RNA hybrids, which we quantified after subtraction of the cytoplasmic signal (Supplementary Figure S4C, D). *MEN1*-KO moderately increased the nuclear RNase H1 signal and the co-localization of RNase H1 with S9.6 in the nucleolus (Supplementary Figure S4E), which suggests that the deletion of *MEN1* leads to the accumulation of R-loops, which appear to recruit RNase H1 into nucleoli. Next, we confirmed the IF results using the more accurate method of DRIP followed by qPCR (DRIP-qPCR). DNA−RNA hybrids accumulated up to at least 1.5-fold more in the *MEN1*-KO cells than in the *MEN1*-WT cells in all analyzed genes; these gene regions have been shown to existence of abundant R-loops (34,57). The hybrid signals were eliminated by RNase H treatment as a confirmation of the specificity of the assay (Figure 4E). Immunoblotting also indicated that *MEN1*-KO modestly augmented the chromatin abundance of RNase H1 but reduced the enrichment of MBNL1 [a splicing factor that binds a stem–loop structure within cardiac troponin T pre-mRNA (58)] and DDX17, an RNA helicase that unwinds DNA–RNA hybrids (59) in NCI-H460 cells with or without Mad treatment (Supplementary Figure S4F). These results suggest that *MEN1* precludes the formation of R-loops presumably via an RNA helicase-dependent mechanism in addition to affecting the nucleolar–nucleoplasmic distribution of RNase H.

As expected, R-loops formed upon the deletion of *MEN1* caused robust DNA damage, as evidenced by the increased signal for the phosphorylation of histone variant H2AX (γH2AX), a marker of DNA damage (Figure 4F–I; Supplementary Figure S4G, H) (60). IF staining revealed that *MEN1* KO led to a prominent increase in the amounts of 53BP1 foci, an effector of the DSB response (61), and elevated the co-localization of 53BP1 with γH2AX in MEFs treated with aphidicolin (APH), an inhibitor of DNA polymerase that induces DNA breaks within chromatin fragile sites (Figure 4F, G) (62). Treatment with Mad resulted in time-dependent activation of γH2AX in NCI-H460 cells and MEFs, and this effect was enhanced by *MEN1* deficiency (Figure 4H, I; Supplementary Figure S4G, H). Importantly, overexpression of RNase H1 in *MEN1*-KO cells markedly reduced the level of γH2AX compared with that in *MEN1*-KO cells (Figure 4H, I), which suggests that the increase in DNA damage in *MEN1*-deleted cells is R-loop dependent. Immunoblotting confirmed that *MEN1* deletion markedly enhanced chromatin levels of γH2AX and phosphorylated ATM (p-ATM), a canonical DDR pathway (Supplementary Figure S4F) (63). In contrast, overexpression of *MEN1* in A549 cells markedly attenuated γH2AX activation induced by treatment with APH compared with in the vector cells (Supplementary Figure S4I, J). To test whether the inactivation of *MEN1* generated DSBs or activated DDR signaling by some alternative pathway, we carried out a neutral comet assay and observed that the comet tail moment in *MEN1*-KO cells treated with or without APH was significantly increased, and this effect was suppressed by RNase H1 overexpression (Figure 4J; Supplemental Figure S4K), which provides direct evidence that *MEN1* deficiency ultimately contributes to DSB accumulation and confirms that DNA damage generated in *MEN1*-deleted cells is R-loop dependent.

Our previous studies showed that the loss of *MEN1* gave rise to DDR activation and lung tumorigenesis (21). To elucidate the correlation between menin expression and R-loop-mediated DNA damage and genome instability in human lung cancer samples, we randomly collected 52 lung cancer specimens, histologically classified samples from 42 patients with LUAD, 8 patients with squamous carcinoma and 2 patients with lung neuroendocrine carcinoma, and corresponding adjacent non-cancerous specimens (Supplementary Table S1). Our results showed that lung cancer tissues possessed dramatically reduced levels of menin expression and increased γH2AX expression compared with the adjacent non-cancerous tissue (Supplementary Figure S4L–N). These human lung cancer tissues consistently showed abundant accumulation of R-loops, as confirmed by increased IF staining for the S9.6 antibody (Supplementary Figure S4O, P). Moreover, we observed that chromosomal instability markers, such as nuclear buds (NBUDs), nucleoplasmic bridges (NPBs) and micronuclei (MNi), were increased in *Men1*^Δ/Δ^ MEFs (Figure 4K, L). Taken together, these findings indicate that *MEN1* protects cells from R-loop-mediated DNA damage and chromosomal instability during lung tumorigenesis.

### *MEN1*-regulated DNA damage-mediated AS is involved in lung cancer

**Figure 5.**
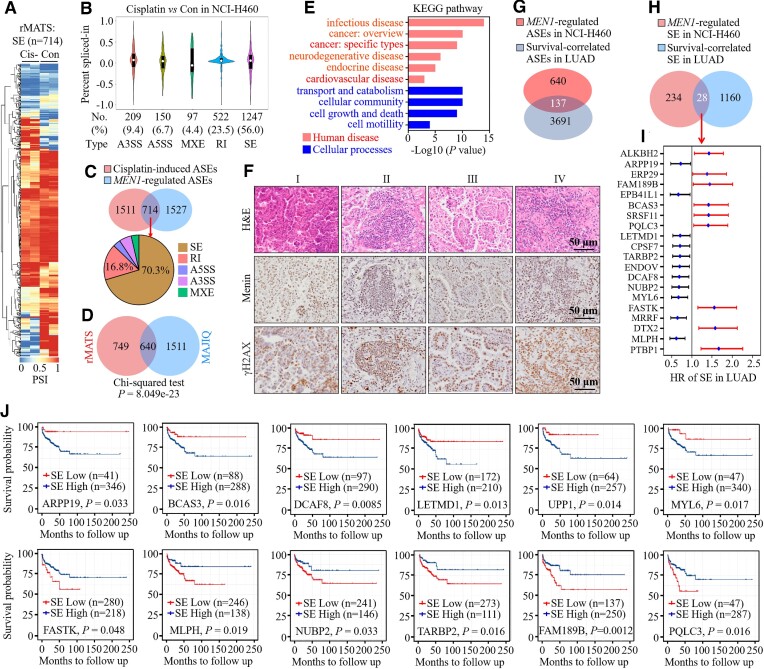
*MEN1*-regulated DNA damage-mediated AS is involved in lung cancer. (**A**) AS heatmap showing PSI values for differentially spliced SE events between control (Con-) and Cisplatin (Cis-)-treated NCI-H460 cells. (**B**) Dot plot showing the distribution of PSI values for each AS type. (**C**) Venn diagram showing the intersection of Cisplatin-induced ASEs and *MEN1*-regulated ASEs (top). The AS types and their respective distributions are shown (bottom). (**D**) Venn diagram showing the intersection of ASEs between the rMATS and MAJIQ datasets. The *P*-value was determined with the χ^2^ test. (**E**) KEGG pathway enrichment analysis of all genes whose SEs were affected by *Men1* knockout in NCI-H460 cells. (**F**) H&E and IHC staining for the menin and γH2AX proteins in lung cancer specimens at different stages (I, II, III and IV); scale bars, 50 μm. (**G**) Venn diagram showing the intersection of *MEN1*-regulated ASEs in NCI-H460 cells and survival-correlated ASEs in LUAD. (**H**) Venn diagram showing the intersection of *MEN1*-regulated SEs in NCI-H460 cells and survival-correlated SEs in LUAD. (**I**) Hazard ratio (HR) of the top 20 *MEN1*-regulated SEs and survival-correlated SEs from (H). (**J**) LUAD patients were divided into two groups with high and low PSIs of SEs, and Kaplan−Meier survival curves were drawn between the two groups for each SE from (H).

We next evaluated whether *MEN1* dysfunction causes aberrant AS patterns through R-loop-induced activation of DNA damage. We reanalyzed RNA-seq in *MEN1*-WT and *MEN1*-KO NCI-H460 cells after Cisplatin treatment. As expected, Cisplatin treatment resulted in changes in gene expression profiles, in which 168 genes were down-regulated and 461 genes were up-regulated (Supplementary Figure S5A; Supplementary Data S5). Further rMATS analysis of the Cisplatin-treated *MEN1*-WT cells revealed aberrant RNA splicing profiles of 2225 differential splicing events for 1838 genes, with 56% and 23.5% of the events being SEs and RIs, respectively (Figure 5A, B; Supplementary Data S6). These data indicate that activation of DNA damage leads to aberrant AS patterns and transcription profiles. Similar to what we observed in the lung tissue of *Men1*-deficient mice, knocking out *MEN1* NCI-H460 cells resulted in an obvious change in AS patterns and transcription profiles (Supplementary Figure S5B, C). We identified 2241 ASEs (corresponding to 1845 genes) that were differentially affected by *MEN1* deletion (Supplementary Figure S5D). The distribution of the ASE types is illustrated in Supplementary Figure S5E; most of these ASEs were SEs (62.2%). We also found that ∼22% of DEGs (408 genes) underwent two or more types of AS; one gene may even have had up to four types of AS affected by *MEN1* depletion (Supplementary Figure S5F). Moreover, we identified 1228 DEGs in the *MEN1*-KO cells compared with the *MEN1*-WT cells, and only 9.2% of these DEGs (113 genes) were found in the ASEs (Supplementary Data S3); among them, 34.5% (39 genes) were up-regulated and 65.5% (74 genes) were down-regulated. However, no significant correlation between gene expression and their PSI was found for any of the 113 genes (data not shown), which further suggests that the alteration in AS patterns caused by *MEN1* deletion in mouse lung tissue or lung cancer cells is not a direct result of changes in transcription.

Unexpectedly, the incidence of *MEN1* deletion-induced ASEs (2241 events between the KO and WT cells) in the untreated *MEN1*-WT and *MEN1*-KO cells was nearly equal to that after Cisplatin treatment of the *MEN1*-WT cells (2225 events between the Cisplatin- and Con-treated cells) (Figure 5C, top). A total of 714 of the 2241 *MEN1*-regulated ASEs overlapped with the Cisplatin treatment-induced ASEs, and the majority (70.3%) of the 714 common events were SE events (Figure 5C, bottom), which were mainly involved in RNA processing, regulation of RNA splicing, DNA repair, base-excision repair and cell cycle progression (Supplementary Figure S5G). These findings are consistent with previous studies demonstrating that DNA damage triggered splicing pattern reprogramming of transcripts of genes pivotal for DDR and the regulation of genome stability (64). The incidence of *MEN1* deletion-related ASEs was not further increased by Cisplatin treatment (1655 differential splicing events were detected in the Cisplatin-treated *MEN1*-WT and *MEN1*-KO cells) (Supplementary Data S7). These analyses demonstrated that inactivation of *MEN1* induced concerted reprogramming of AS that is partially dependent on the DNA damage activation.

Given that *MEN1* plays an important role in the regulation of RNA metabolism and genome stability, we next investigated the biological implications of *MEN1*-regulated ASEs in human lung cancer. By analyzing overlapping ASEs between rMATS and MAJIQ in NCI-H460 cells, we identified 640 high-confidence differentially spliced genes that were regulated upon *MEN1* deletion (Figure 5D). KEGG pathway enrichment analysis confirmed that these differentially spliced genes were mainly involved in various human diseases, especially cancers, and cellular processes (cell growth and death and cell motility) (Figure 5E). Consistent with these results, the higher the tumor stage, the lower the menin expression in lung cancer specimens (Figure 5F); the menin expression was inversely correlated with tumor size and tumor stage, but there were no significant correlations between menin expression and sex or other clinicopathological characteristics (Supplementary Figure S5H; Supplementary Table S1). Reciprocally, the levels of DNA damage in human lung cancer samples increased gradually with disease progression (Figure 5F; Supplementary Figure S5I). Furthermore, we took advantage of data analyzed by others who generated AS profiles in LUAD and identified a total of 3691 survival-associated ASEs by conducting univariate survival analyses for OS (65). Using Venn diagram analysis, we found that 137 out of 640 *MEN1*-related ASEs identified in NCI-H460 cells overlapped with LUAD survival-associated ASEs (Figure 5G). Approximately 37% of the ASEs (234 events) were SE events, of which 28 SE events were found among LUAD survival-associated SE events (Figure 5H); 71% (20/28) of the SE events were validated to be correlated with the OS of LUAD patients (of these SE events, 9 predicted significantly worse outcomes and 11 predicted good outcomes) (Figure 5I). In addition, we downloaded clinical parameters of the LUAD cohort from the TCGA database and divided the patients into high- and low-PSI groups on the basis of the median PSI of 28 *MEN1*-regulated SE genes. A survival analysis showed that 17 out of 28 *MEN1*-regulated SE events also correlated with patient OS, of which 8 SE events showed a negative correlation and 9 SE events displayed a positive correlation (Figure 5J). Interestingly, however, the mRNA expression of these genes did not correlate with the OS of patients (Supplementary Figure S5J), which suggests that *MEN1-*regulated gene AS, rather than their gene expression, is dramatically correlated with LUAD patient survival. Indeed, RT–PCR results indicated that the deletion of *MEN1* in lung cancer cells increased the instances of short splicing isoforms in the *FAM189B*, *NUBP2*, *ENDOV* and *TARBP2* genes, but inhibited generation of the *CPSF7*, *MRRF* and *LETMD1* splicing isoforms (Supplementary Figure S5K). Altogether, these findings further support the claim that *MEN1*-regulated ASEs have functional significance in lung cancer.

### *MEN1* deficiency sensitizes human lung cancer cells to splicing inhibitors

**Figure 6.**
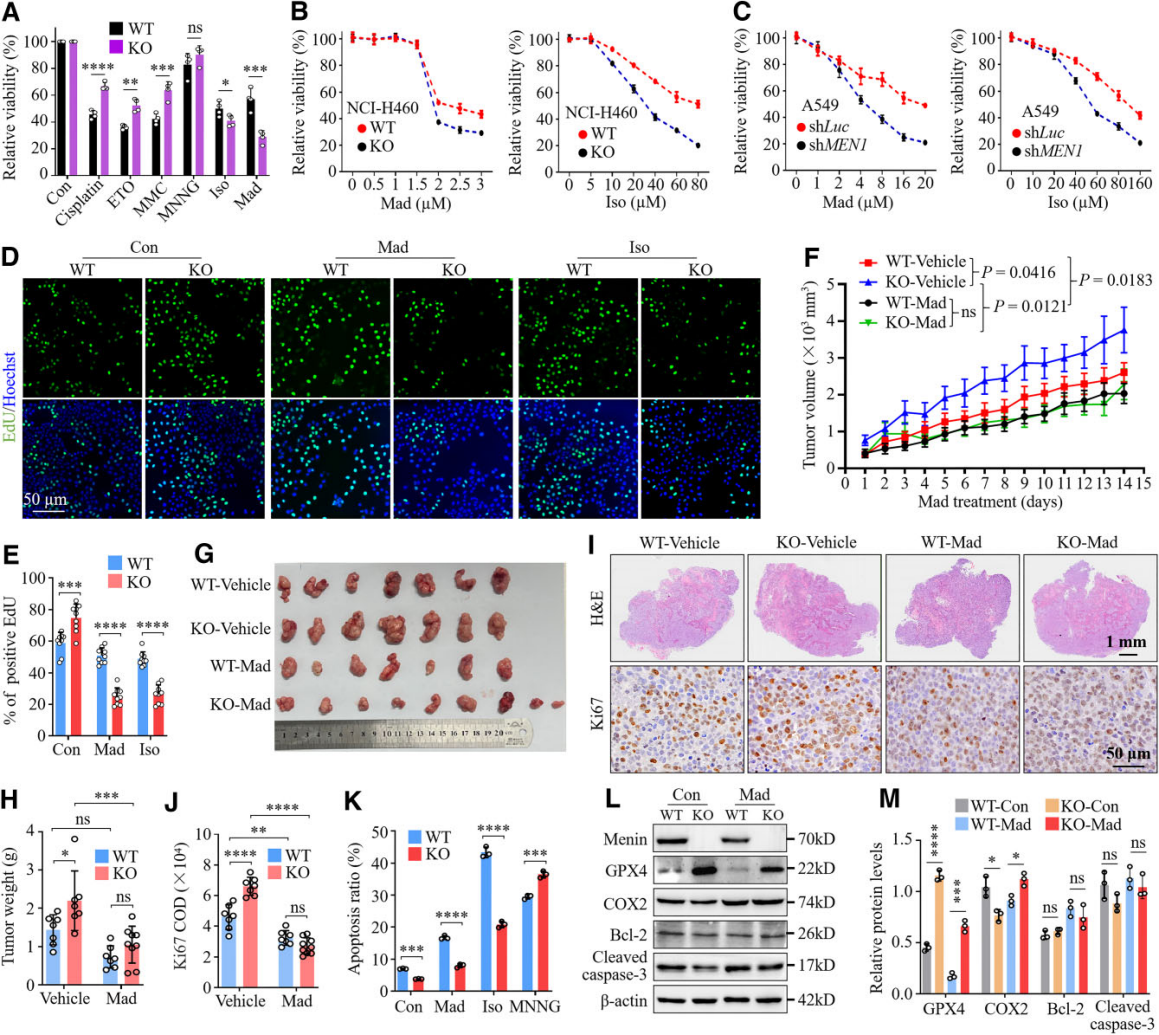
*MEN1* deficiency sensitizes human lung cancer cells to splicing inhibitors. (**A**) Relative cell viability of *MEN1*-WT and *MEN1*-KO NCI-H460 cells at 48 h after treatment with 2 μM Cisplatin, 0.2 mg/ml ETO, 6 μM MMC, 5 μM MNNG, 2 μM Mad or 20 μM Iso. The relative cell viability in each group was normalized to the level of its control (Con) as 100%. (**B** and **C**) Relative cell viability of *MEN1*-WT and *MEN1*-KO NCI-H460 cells (B) and sh*Luc*- and sh*MEN1-*A549 cells (C) under the indicated concentrations of Mad or Iso for 48 h. The relative cell viability in each group was normalized to the level of its untreated group as 100%. (**D**) EdU (green) and Hoechst (blue) staining of *MEN1*-WT and *MEN1*-KO NCI-H460 cells 48 h after treatment with 2 μM Mad or 20 μM Iso; scale bar, 50 μm. (**E**) Quantification of EdU-positive cells for the experiments in (D). Dots in the histogram depict individual images. Data are represented as the mean ± SD (*n* = 9 images), analyzed by two-tailed unpaired *t*-test; ***P < 0.001; ****P < 0.0001. (**F**) *In vivo* growth of *MEN1*-WT and *MEN1*-KO NCI-H460 cell xenograft tumors. Mice received daily i.p. injections of DMSO (vehicle) or Mad (3 mg/kg body weight). Mad treatment was withdrawn on day 12, and tumor volumes were measured until the end of the experiment. (**G**) Bright-field images of *MEN1*-WT and *MEN1*-KO NCI-H460 xenograft tumors dissected from mice following the last measurement of tumor volumes. (**H**) Quantification of tumor weights of dissected *MEN1*-WT and *MEN1*-KO NCI-H460 xenograft mice on day 14 of Mad treatment. (**I**) Representative H&E (top) and Ki67 IHC staining (bottom) images of tumor sections from *MEN1*-WT and *MEN1*-KO NCI-H460 xenograft mice at 14 days; scale bars, 1 mm (H&E) and 50 μm (IHC). (**J**) Quantification of Ki67 IHC staining in the indicated xenograft tumor sections for the experiments in (I). (**K**) Quantification of the apoptosis ratio for the experiments in Supplementary Figure S6E. (**L** and **M**) Immunoblotting of the indicated proteins (L) and quantification of relative protein levels (M) in *MEN1*-WT and *MEN1*-KO NCI-H460 cells treated with 4 μM Mad. In (E), (H) and (J), dots in the histogram depict individual samples. Error bars with the mean ± SEM [*n* = 7 mice per group except the KO-Mad group (*n* = 9 mice)], analyzed by one-way ANOVA; *P < 0.05; **P < 0.01; ***P < 0.001; ****P < 0.0001; ns, not significant.

We further investigated whether splicing inhibitors can reverse the malignant behaviors of *MEN1*-deficient lung cancer cells. We tested two splicing inhibitors (Mad and Iso), three anticancer drugs (Cisplatin, ETO and MMC) and MNNG for selective lethality in *MEN1*-WT and *MEN1*-KO NCI-H460 cells. The six tested chemical drugs tested inhibited the proliferation of NCI-H460 cells (Figure 6A; Supplementary Figure S6A, B). Interestingly, once a certain concentration was reached, both Mad and Iso, but not Cisplatin, ETO or MMC, suppressed *MEN1*-KO cells to a greater extent than *MEN1*-WT cells and did so in a dose-dependent manner; MNNG, to a lesser extent, also exhibited selective lethality in *MEN1*-KO cells (Figure 6A, B). A similar phenomenon was found in Mad- and Iso-treated *MEN1* knockdown A549 cells (Figure 6C) and *Men1*-deleted MEFs (Supplementary Figure S6C), which highlights the selective effect of splicing inhibitors in inducing the death of *MEN1*-deficient lung cancer cells (Supplementary Figure S6D). Further EdU cell proliferation analysis showed significantly more EdU-positive cells in the *MEN1*-KO cells than in the *MEN1*-WT cells; exposure to Mad or Iso resulted in a significant decrease in EdU-positive cells and showed higher selectivity in *MEN1*-KO cells (Figure 6D, E). These observations indicate that *MEN1* deficiency sensitizes cells to growth inhibition induced by splicing inhibitors.

To delineate the selective suppression of splicing inhibitors in *MEN1*-deficient lung cancer growth *in vivo*, we subcutaneously transplanted *MEN1*-WT and *MEN1*-KO NCI-H460 cells into nude mice. The deletion of *MEN1* profoundly promoted tumor growth for the duration of the observation period (Figure 6F). As expected, treatment with Mad inhibited the growth of *MEN1*-deficient tumors more efficiently than WT-*MEN1*-expressing tumors (Figure 6F). The mice were sacrificed on day 14, and the tumor weight and Ki67 index of the *MEN1*-KO-vehicle xenograft tumors were dramatically augmented compared with these measures in the *MEN1*-WT-vehicle xenograft tumors; treatment with Mad reduced the tumor weight and Ki67 index, and showed higher selectivity for the *MEN1*-KO xenograft tumors (Figure 6F–J). These findings indicate that splicing inhibitors such as Mad and Iso predominantly suppress the growth of lung tumors in which *MEN1* is dysfunctional.

Finally, we sought to define the modality of *MEN1* deletion-related cell death induced by splicing inhibitors. Annexin-V/FITC staining and flow cytometry showed that both Mad and Iso induced cell apoptosis but with no selective lethality in the *MEN1*-WT and *MEN1*-KO cells, whereas MNNG-induced cell apoptosis exhibited exceptional selectivity for the *MEN1*-KO cells (Figure 6K; Supplementary Figure S6E). These data suggest that Mad and Iso selectively inhibit the proliferation of *MEN1*-KO cells independent of the apoptosis pathway. We were surprised to find that *MEN1* deletion significantly suppressed ferroptosis, a non-apoptotic form of cell death (66), as confirmed by increased GPX4 expression and decreased COX2 expression, effects that were rescued by Mad or Iso treatment (Figure 6L; Supplementary Figure S6F). Moreover, the fold change in GPX4 and COX2 expression induced by Mad or Iso in the *MEN1*-KO cells was higher than that in the *MEN1*-WT cells (Figure 6M; Supplementary Figure S6G), whereas this discrepancy was not observed in a comparison of Bcl-2 and cleaved caspase-3 expression (Figure 6L, M). Taken together, these results demonstrate that Mad and Iso exhibit selective growth inhibition in *MEN1*-deficient lung tumors, which may be explained by ferroptosis rather than increased apoptosis.

## DISCUSSION

Dysregulation of AS substantially contributes to an ever-increasing number of human diseases. However, the regulation of alternative pre-mRNA splicing processes is poorly understood. In the present study, we provide multiple lines of evidence supporting *MEN1* as an important modulator of the AS process, the most important of which is that the deletion of *MEN1* leads to aberrant AS profiles in mouse lung tissue, human lung cancer cells and human breast cancer cells (data not shown). Our subsequent data indicate that *MEN1* slows the rate of Pol II elongation and is required to prevent of R-loop formation and the accumulation of DNA damage. In this connection, because of their augmented chromosomal instability and correlation with LUAD patient survival, lung cancers harboring *MEN1* inactivation may be dependent on a fast Pol II elongation rate to generate R-loops, abundantly accumulating DSBs, thereby supporting tumor growth, which to some extent explains the selective lethality of lung cancer cells to splicing inhibitors. In accordance with this hypothesis, deficiency in *MEN1* provoked the splicing isoform generation, genome instability and cell proliferation. This observation is in line with previous studies showing that highly processive transcription favors exon skipping, whereas DNA damage-inhibited Pol II elongation provokes exon inclusion and tumorigenesis (14).

Because menin lacks motifs that are homologous to known proteins, it is challenging to discover its biochemical function. Menin has been widely characterized as an important scaffold protein that regulates gene transcription by interacting with multiple proteins with diverse functions (22). Consistent with this conception, menin activates gene transcription by binding to the transcription activator MLL1; in contrast, the menin–JUND interaction suppresses JUND-induced transcription (45). Here we show that, in addition to having this quantitative effect, *MEN1* also impacts the quality of transcripts by altering exon skipping in the pre-mRNAs of some genes. We and others have previously demonstrated that *MEN1* inactivation or deletion leads to various human diseases, such as renal fibrosis (67), diabetes (68) and lung cancers (21). Since the AS program tightly controls the quantity and quality of post-transcriptional gene expression, which plays a crucial role in diverse cellular processes, including cell proliferation, differentiation and death, we therefore reason that these defects are caused in part by abnormal AS programs upon *MEN1* dysfunction. The following evidence supports this assumption: (i) abundant differential AS events, instead of a small number of DEGs, are strongly influenced upon *MEN1* knockout in mouse lung tissue and human cancer cells; (ii) menin extensively binds to CD44 variant exons and reduces their abundance by slowing Pol II elongation; and (iii) in *MEN1*-regulated SE events, RNA splicing isoform levels, rather than their corresponding transcription levels, correlate with LUAD patient survival (Figure 5J; Supplementary Figure S5J).

On a mechanistic level, *MEN1* maintains homeostasis of the AS network in part by slowing down the rate of RNA polymerase elongation. Alternative RNA splicing processes are tightly coupled to Pol II transcription (69). This is an important mechanism for controlling the AS of the vast majority of genes. In this study, we showed that menin affects AS patterns by decreasing the rate of Pol II elongation. Under these circumstances, slowing down Pol II elongation favors the use of weak or suboptimal splice sites by increasing the time window opportunity for their recognition by splicing factors before downstream stronger splice sites are synthesized (47). Our findings propose that menin facilitates pausing of Pol II elongation on the DNA template, as exemplified by the oncogene *CD44* or *c-Myc*, which may lead to Pol II interaction with key splicing factors through the CTD of the large subunit of Pol II; its exact mechanisms are currently being investigated. Moreover, although menin has been shown to be a DNA-binding protein that plays a significant role in gene expression (23), whether it directly regulates AS by binding to target RNA sequences has not been explored. Our unpublished RIP-seq data analysis showed that menin is capable of directly binding to a subset of RNAs. Considering the fundamental role of RNA-binding proteins in regulating the choice of splice site during the AS process (26,70), we propose that menin directly regulates RNA splicing through its direct interplay with specific RNAs under both physiological conditions and exposure to external stimuli. However, how the function of the menin RNA-binding protein impacts alternative pre-mRNA splicing remains to be determined. All these findings indicate that menin is a crucial splicing regulatory factor that equilibrates the AS network through indirect Pol II elongation-dependent and direct RNA binding-dependent mechanisms.

In conclusion, we reveal a complex relationship among *MEN1* dysfunction, abnormal AS, R-loop formation, the accumulation of DNA damage and genome instability during lung tumorigenesis. *MEN1* inactivation accelerates the rate of Pol II elongation, producing abundant nascent pre-mRNAs. This enables unspliced nascent transcripts to be retained on template chromatin and triggers R-loop formation and DSB accumulation, thereby leading to genome instability and lung cancer progression. Given the roles of these changed *MEN1*-related exon skipping events in the progression of lung cancer, their potential as therapeutic targets specifically against *MEN1*-deficient cancer should be investigated in the future.

## Supplementary Material

gkad548_Supplemental_Files

## Data Availability

The antibodies, primers, cell lines, lung cancer samples, mice, plasmids, chemicals, drugs and commercial kits used in this study are listed in Supplementary Tables S2 and S3. RNA-seq data in this study were deposited in the National Center for Biotechnology Information (NCBI) under the accession numbers PRINA888804 for the *Men1*^f/f^ and *Men1*^Δ/Δ^ mouse lung tissue and PRINA888457 for the *MEN1*-WT and *MEN1*-KO NCI-H460 cells. Information on the lung cancer patients is available as Supplementary Data S8. Datasets for the 3691 LUAD survival-associated AS events are available from published references (65). All other data supporting the findings of this study are available from the corresponding author on reasonable request. Source data are provided with the paper.
