# Supplementary material for: MEN1 is a regulator of alternative splicing and prevents R-loop-induced genome instability through suppression of RNA polymerase II elongation
Source: Nucleic Acids Res. 2023 Jul 3;51(15):7951–71. doi: 10.1093/nar/gkad548 (PMC10450199; doi:10.1093/nar/gkad548)
Supplement: gkad548_Supplemental_Files [file gkad548_supplemental_files.zip › Supplementary information.docx]

**Supplementary information**

***MEN1* is a regulator of alternative splicing and prevents R-loop-induced genome instability through suppression of RNA polymerase II elongation**

Bangming Jin^1,2,3,4,^^†,^*, Jiamei Zhu^1,3,4,†^, Ting Pan^1,3,4,†^, Yunqiao Yang^1,2,3,4,†^, Li Liang^1,3,4^, Yuxia Zhou^1,3,4^, Tuo Zhang^1,3,4^, Yin Teng^2,5^, Ziming Wang^1,3,4^, Xuyan Wang^1,3,4^, Qianting Tian^3,5^, Bing Guo^1,3,4^*, Haiyang Li^2,5^*, Tengxiang Chen^1,2,3,4,^*

^1^Department of Physiology, School of Basic Medical Sciences, Guizhou Medical University, 550025 Guiyang, China

^2^Department of Surgery, Affiliated Hospital of Guizhou Medical University, 550025 Guiyang, China

^3^Transformation Engineering Research Center of Chronic Disease Diagnosis and Treatment, Guizhou Medical University, Guiyang, China

^4^Guizhou Provincial Key Laboratory of Pathogenesis and Drug Research on Common Chronic Diseases, Guizhou Medical University, 550025 Guiyang, China

^5^Guizhou Institute of Precision Medicine, Affiliated Hospital of Guizhou Medical University, 550025 Guiyang, China

^†^These authors contributed equally to this work.

*To whom correspondence should be addressed. Tel: +86 0851 88174007 or +86 15811034377; Fax: +86 0851 88416078; E-mail: guobingbs@126.com; BMJin@gmc.edu.cn; lihaiyang@gmc.edu.cn; txch@gmc.edu.cn

**
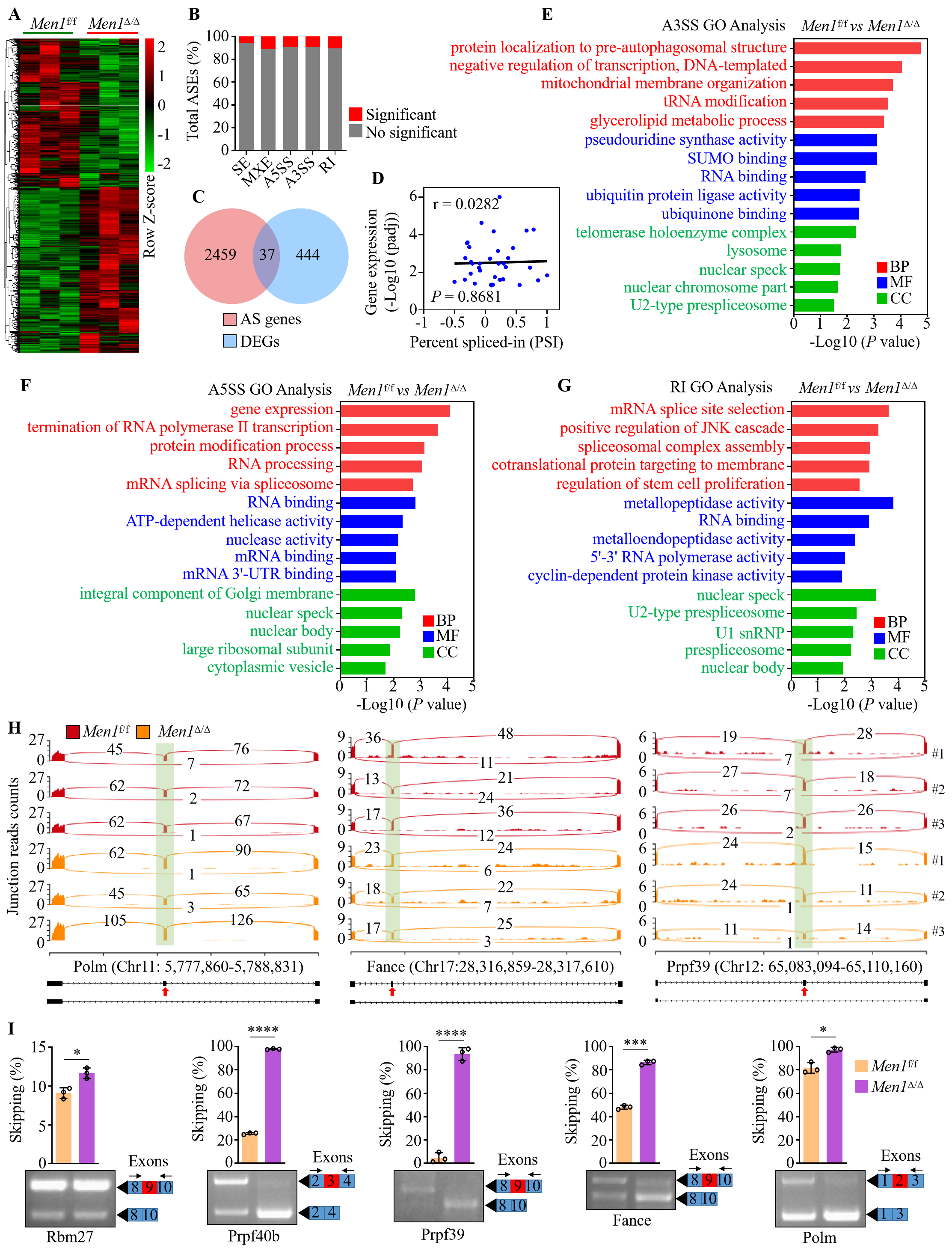
**

**Supplementary Figure 1.** ***MEN1* deficiency disrupts global alternative pre-mRNA splicing profiles. (A)** Heatmap visualization of 444 DEGs (*P* < 0.05 and fold change ≥ 1.5) between *Men1*^f/f^ and *Men1*^Δ/Δ^ mice lung tissue. n = 3 biological replicates per group; Z-score-normalized FPKM values were determined based on independent biological samples with different RNA-seq data. **(B)** Bar plot of the ASEs observed in RNA-seq analysis of *Men1*^f/f^ and *Men1*^Δ/Δ^ mice lung tissue. **(C)** Venn diagram showing overlap of the AS genes and DEGs in *Men1*^f/f^ and *Men1*^Δ/Δ^ mice lung tissue. **(D)** Correlation analysis between the PSI of differentially spliced genes and corresponding gene expression in *Men1*^f/f^ and *Men1*^Δ/Δ^ mice lung tissue. Spearman's correlation coefficient and *P* value by Spearman’s test is indicated. **(E-G)** GO enrichment analysis of all genes whose A3SS, A5SS and RI are respectively affected by *Men1* loss in mouse lung tissue. **(H)** Sashimi plot showing the increased skipped exon of the *Plom*, *Fance*, and *Prpf39* genes in *Men1*^Δ/Δ^ mice lung tissue compared to those of *Men1*^f/f^ mice. Green highlights indicate AS events, with the number of junction reads indicated for each event. **(I)** RT‒PCR and gel electrophoresis analysis of the indicated SE genes in *Men1*^f/f^ and *Men1*^Δ/Δ^ MEFs (bottom); Quantification of skipping rates of the indicated SE genes (top). Information for target exons is shown in the right panel. Blue box, constitutive exon; red boxes, skipped exon; the images are representative of three independent experiments; data are represented as the mean ± SD (n = 3 biologically independent experiments), analyzed by two-tailed unpaired *t* test; **P* < 0.05; ***P* < 0.01; ****P* < 0.001; *****P* < 0.0001.


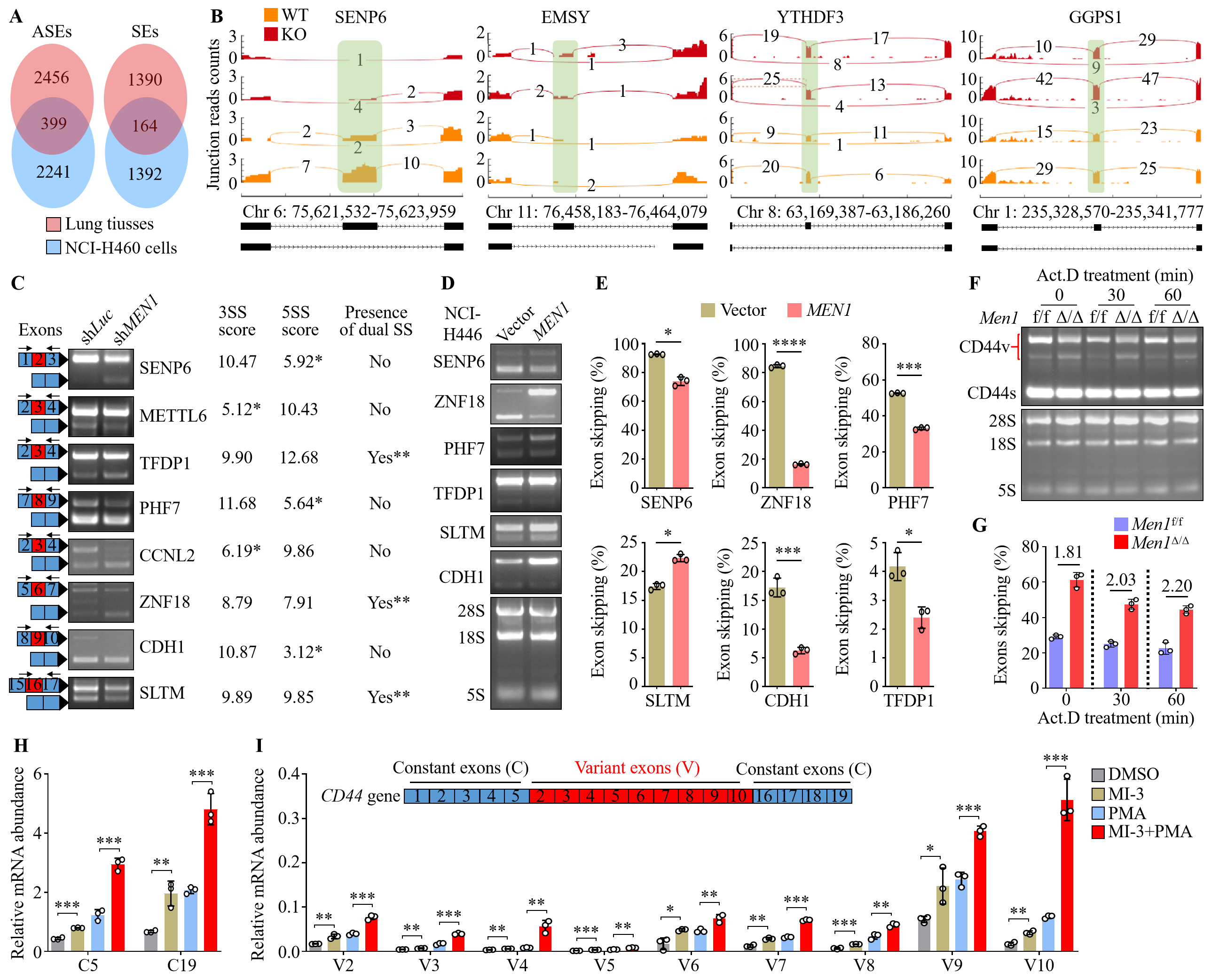


**Supplementary Figure 2. *MEN1* alters exon skipping and the abundance of RNA splicing isoforms. (A)** Venn diagram showing the overlap of *MEN1*-regulated ASEs (left) and *MEN1*-regulated SEs (right) of lung tissue and NCI-H460 cells. **(B)** Sashimi plot showing the changed exon-skipping of the *SENP6*, *EMSY*, *YTHDF3*, and *GGPS1* genes in *MEN1*-KO NCI-H460 cells compared to that in *MEN1*-WT cells. Green highlights indicate AS events, with the number of junction reads indicated for each event. **(C)** left, RT-PCR and gel electrophoresis analysis of the indicated SE genes in sh*Luc* and sh*MEN1* A549 cells. Information for target exons is shown on the left panel. Blue box, constitutive exon; red boxes, skipped exon; right, feature analysis for splice sites of the *MEN1*-regulated SE genes. 5SS and 3SS motif scores were calculated, and the presence of dual-splice sites were determined by the matrices of splice sites available in ESEfinder version 3.0. The threshold score of 5SS and 3SS is 6.67 and 6.632, respectively. *, scores less than threshold; **, splice sites that can be recognized as both 5SS and 3SS. **(D)** RT-PCR and gel electrophoresis analysis of the indicated SE genes in control (vector) and *MEN1*-overexpressing (*MEN1*) NCI-H460 cells. **(E)** Quantification of skipping rates of the indicated SE genes for the experiments in **D**. **(F)** RT-PCR and gel electrophoresis analysis of CD44 variant exons (CD44v) in *Men1*^f/f^ and *Men1*^Δ/Δ^ MEF cells treated with 2 μg/mL Act.D for indicated time. Bottom gel shows total RNA in the samples. **(G)** Quantification of skipping rates of the CD44v for the experiments in **F**. The numbers in the figure represent the ratio of skipping rates of *Men1*^Δ/Δ^ cells to that of *Men1*^f/f^ cells. **(H, I)** qPCR was used to detect the mRNA abundance of CD44 constant (C) and variant exons (V) in A549 cells treated with 20 μM MI-3 or 40 ng/mL PMA or both combination for 72 h. In **C**, **D**, and **F**, images are representative of three independent experiments. In **E**, **G**, **H**, and **I**, data are represented as mean ± SD (n = 3 biologically independent experiments), analyzed by two-tailed unpaired *t*-test; **P* < 0.05; ***P* < 0.01; ****P* < 0.001; *****P* < 0.0001.

**
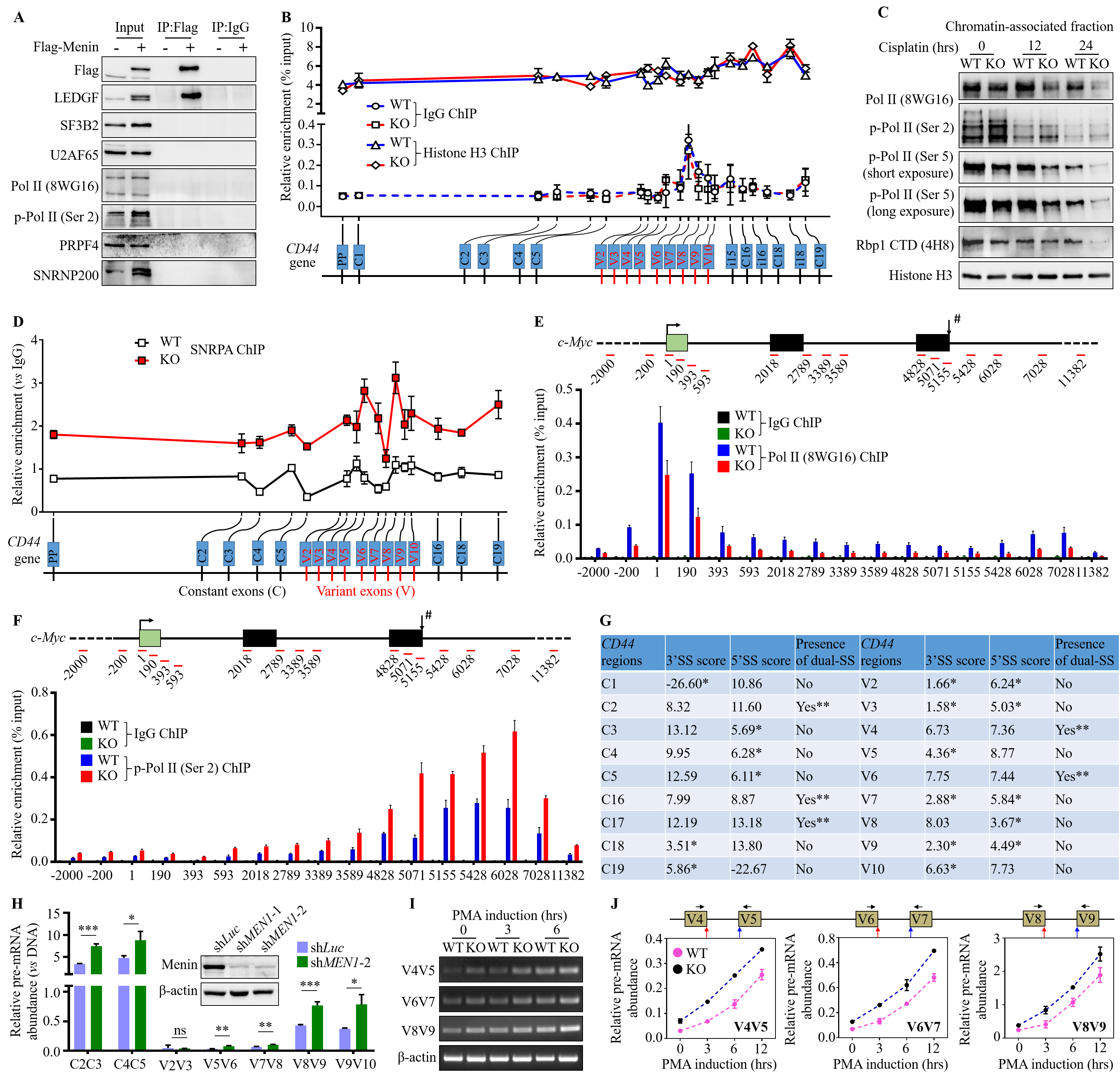
**

**Supplementary Figure 3. *MEN1* regulates AS by the slowing Pol II elongation rate. (A)** Total cellular proteins were extracted from vector and *MEN1*-A549 cells, and Co-IP was performed with 3 × Flag or IgG antibodies, followed by immunoblotting for the indicated proteins. **(B)** Chromatin was extracted from *MEN1*-WT and *MEN1*-KO NCI-H460 cells, and ChIP walking assays were performed with histone H3 or IgG antibodies, followed by qPCR to detect the enrichment of these proteins on the *CD44* gene locus. Amounts of histone H3 are expressed as percentage of the input. **(C)** Chromatin-associated fractions were isolated from *MEN1*-WT and *MEN1*-KO NCI-H460 cells treated with 4 μM Cisplatin for different times, followed by immunoblotting for the indicated proteins. **(D-F)** Chromatin was extracted from *MEN1*-WT and *MEN1*-KO NCI-H460 cells and ChIP walking assays were performed with antibodies against SNRPA (**D**), Pol II (8WG16) (**E**) or p-Pol II (Ser 2) (**F**), followed by qPCR to detect the enrichment of these proteins on the *CD44* or *c-Myc* gene locus. Relative enrichment is expressed as percentage of the input. #, indicates poly(A) signal at +8,570 of the *c-Myc* gene. **(G)** Feature analysis for the splice sites of the *CD44* gene locus. The 5SS and 3SS motif scores were calculated, and the presence of dual-splice sites was determined by the matrices of splice sites available in ESEfinder version 3.0. *, scores less than threshold. **, splice sites that can be recognized as both 5SS and 3SS. **(H)** qPCR quantification of CD44 pre-mRNA exons retained on chromatin. Non-cross-linked DNase-treated chromatin from sh*Luc*- and sh*MEN1*-A549 cells was extracted as described in the Methods. The graph displays the mean ± SD of chromatin-associated RNA relative to the DNA in the input (quantified by qPCR). **(I)** RT‒PCR and gel electrophoresis analysis of CD44 variant exons in *MEN1*-WT and *MEN1*-KO NCI-H460 cells treated with 40 ng/mL PMA for different times. **(J)** qPCR was used to quantify the mRNA abundance of CD44 variant exons in *MEN1*-WT and *MEN1*-KO NCI-H460 cells treated with 40 ng/mL PMA for different times. The positions of primers containing 3SS (red arrows) and 5SS (blue arrows) for qPCR are indicated by black arrows (top). In **A**, **C**, and **I**, images are representative of three independent experiments. In **B**, **D-F**, **H**, and **J**, data are represented as mean ± SD (n = 3 biologically independent experiments), analyzed by two-tailed unpaired *t*-test; **P* < 0.05; ***P* < 0.01; ****P* < 0.001; ns, not significant.

**
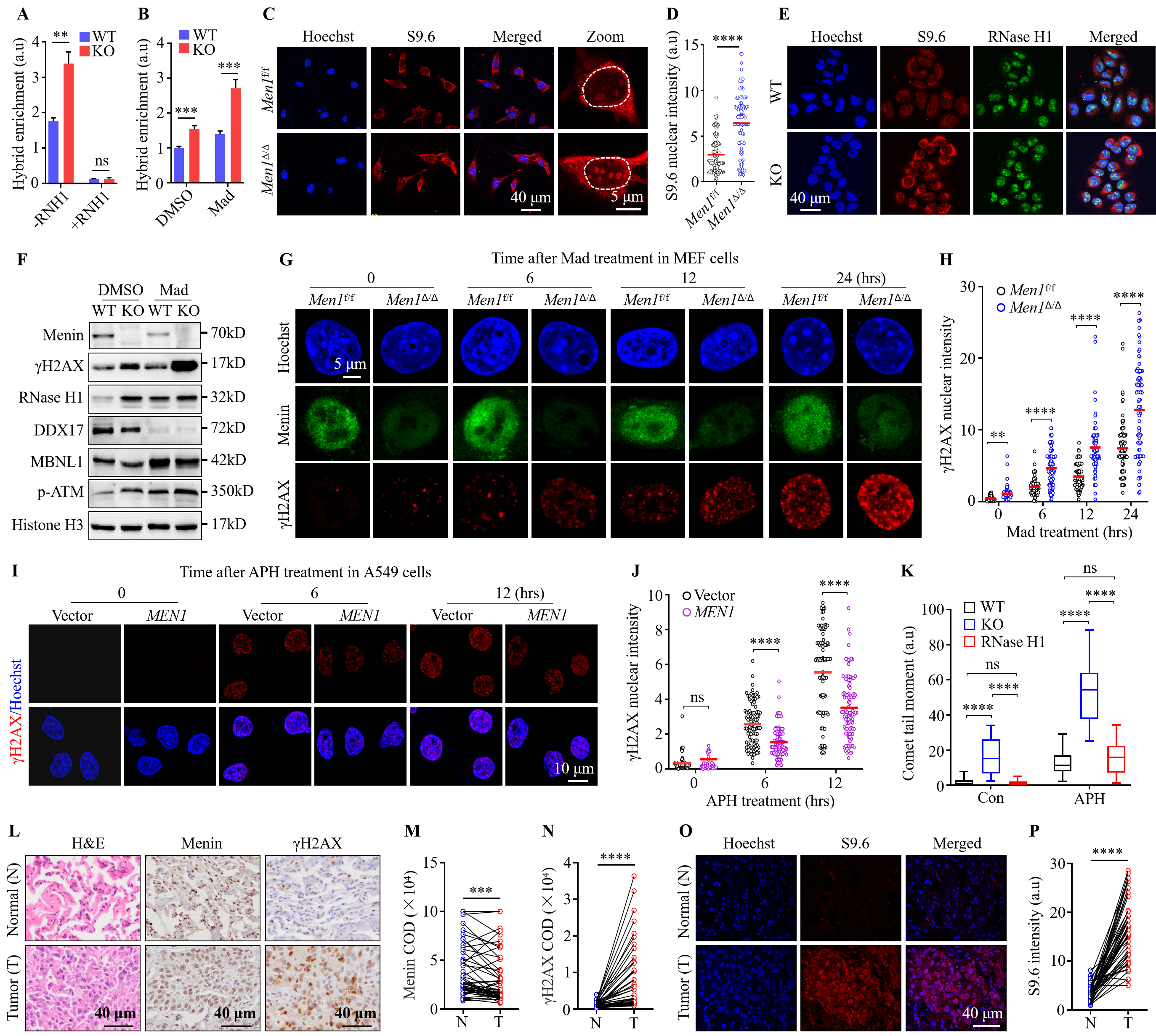
**

**Supplementary Figure 4. *MEN1* prevents R-loop-induced accumulation of DNA damage and genome instability. (A, B)** Quantification of RNA‒DNA hybrids detected by dot blot with the S9.6 antibody for the experiments described in **Figure 4A** (**A**) and **Figure 4B** (**B**). Data are represented as the mean ± SD (n = 3 biologically independent experiments), analyzed by two-tailed unpaired *t* test; ***P* < 0.01; ****P* < 0.001; ns, not significant. **(C**, **D)** IF staining (**C**) and intensity quantification (**D**) of S9.6 in *Men1*^f/f^ and *Men1*^Δ/Δ^ MEFs; scale bars, 40 μm or 5 μm (zoom); a.u, arbitrary units. Dots in the histogram in **D** depict individual cells (n = 96 *Men1*^f/f^ MEFs and n = 104 *Men1*^Δ/Δ^ MEFs). Median values are indicated by red lines (two-tailed Mann-Whitney *U* test; *****P* < 0.0001). **(E)** IF staining of S9.6 (red) and RNase H1 (green) in *MEN1*-WT and *MEN1*-KO NCI-H460 cells; scale bar, 40 μm. **(F)** *MEN1*-WT and *MEN1*-KO NCI-H460 cells were treated with 4 μM Mad for 24 h, and chromatin-associated fractions were isolated, followed by immunoblotting for the indicated proteins. **(G)** *Men1*^f/f^ and *Men1*^Δ/Δ^ MEFs were treated with 4 μM Mad, followed by IF staining for menin (green) and γH2AX (red) antibodies at the indicated time points; scale bar, 5 μm. **(H)** Quantification of γH2AX intensity for the experiment in **G**. Dots in the histogram depict individual cells and more than 100 cells per condition were considered in each experiment. Median values are indicated by red lines (two-tailed Mann-Whitney *U* test; ***P* < 0.001; *****P* < 0.0001). **(I)** Vector and *MEN1*-A549 cells were treated with 0.4 μM APH, followed by IF staining for γH2AX (red) at the indicated time points; scale bar, 10 μm. **(J)** Quantification of γH2AX intensity for the experiments in **I**. Dots in the histogram depict individual cells and more than 100 cells per condition were considered in each experiment. **(K)** Quantification of the comet tail moment for the experiments in **Figure 4J**. More than 150 comets were counted in each experiment, from which a median value was obtained (two-tailed unpaired *t* test; *****P* < 0.0001). **(L)** H&E and IHC staining for the menin and γH2AX proteins in lung cancer (T) and corresponding adjacent noncancerous tissues (N); scale bar, 40 μm. **(M, N)** Quantification of menin (**M**) and γH2AX (**N**) IHC staining in human lung cancer specimens for the experiments in **L**. Dots in the histogram depict individual samples (n = 52 cases); data are represented as the mean ± s.d. (n = 52 cases), analyzed by two-tailed paired *t* test; ****P* < 0.001; *****P* < 0.0001; COD, corrected optical density. **(O)** IF staining of S9.6 (red) in lung cancer (T) and adjacent noncancerous samples (N); scale bar, 40 μm. **(P)** Quantification of S9.6 intensity for the experiments in **O**. Dots in the histogram depict individual samples. Data are represented as the mean ± SD (n = 52 cases), analyzed by two-tailed paired *t* test; *****P* < 0.0001. In **C**, **E**, **F**, G, **I**, **L**, and **O**, images are representative of three independent experiments.

**
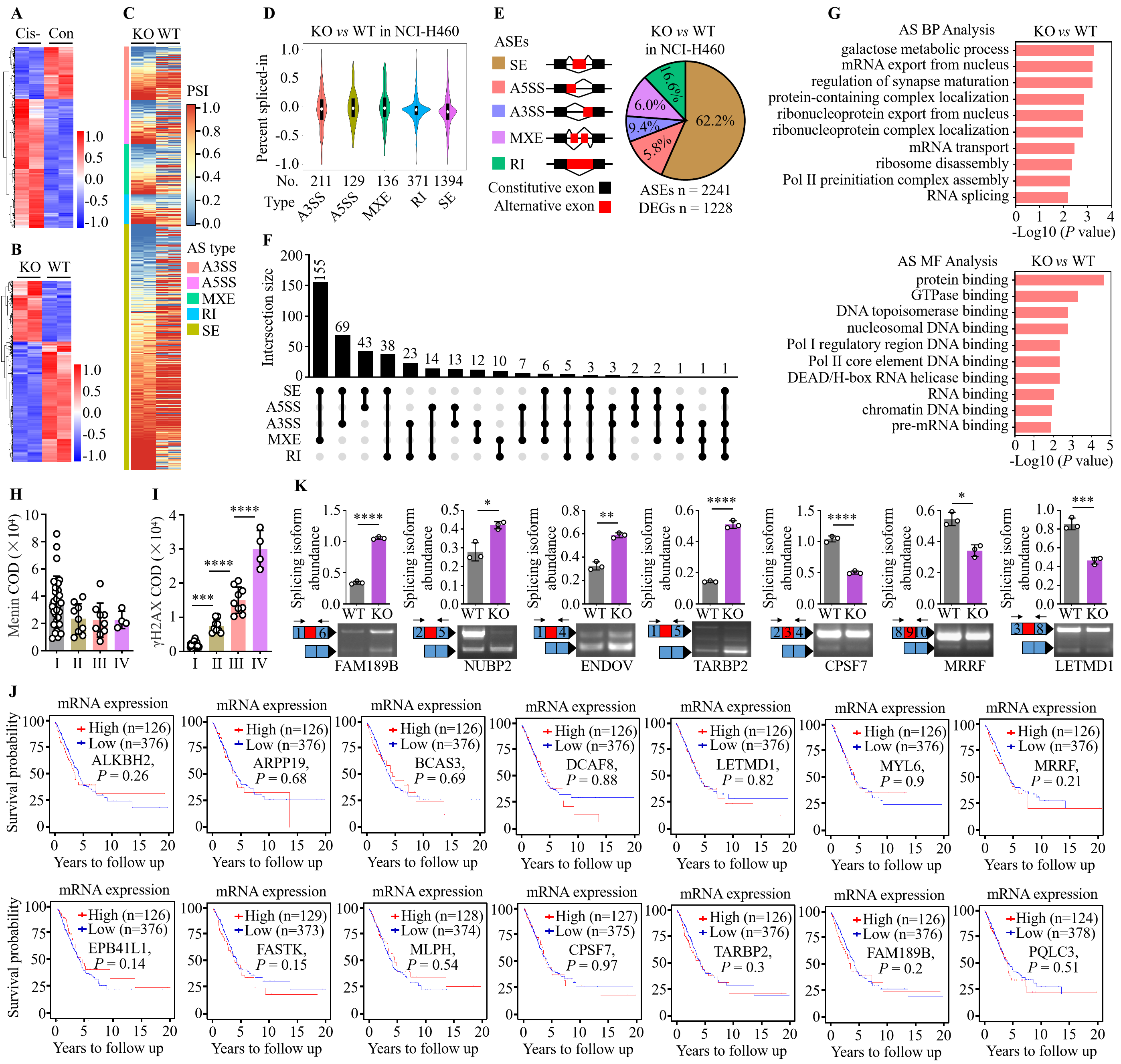
**

**Supplementary Figure 5.** ***MEN1*-regulated DNA damage-mediated alternative splicing is involved in lung cancer. (A, B)** Heatmap visualization of DEGs in Con- and Cisplatin-treated NCI-H460 cells (**A**) and *MEN1*-WT and *MEN1*-KO NCI-H460 cells (**B**). **(C)** Heatmap showing the PSI values of differentially spliced ASEs in *MEN1*-WT and *MEN1*-KO NCI-H460 cells. **(D)** Dot plot showing the distribution of PSI values for each AS type. The PSI between *MEN1*-KO and *MEN1*-WT NCI-H460 cells was based on two independent biological samples from each group. **(E)** Schematic (left) and distribution (right) of five AS types that change upon *MEN1* knockout in NCI-H460 cells. **(F)** UpSet plot of interactions between the five types of *MEN1*-regulated ASEs in NCI-H460 cells. **(G)** GO enrichment analysis showing biological processes (BPs) and molecular functions (MFs) affected by *MEN1*-regulated AS in NCI-H460 cells. **(H, I)** Quantification of menin (**H**) and γH2AX (**I**) IHC staining for the experiments in **Figure 5F**. Dots in the histogram depict individual samples. n = 30, stage I specimens; n = 9, stage II specimens; n = 9, stage III specimens; n = 4, stage IV specimens. Data are represented as the mean ± SD, analyzed by one-way ANOVA; ****P* < 0.001; *****P* < 0.0001; COD, corrected optical density. **(J)** LUAD patients were divided into two groups with high and low expression of transcripts, and Kaplan‒Meier survival curves were drawn between the two groups for each transcript. **(K)** RT‒PCR and gel electrophoresis analysis of the indicated LUAD survival-correlated SE genes in *MEN1*-WT and *MEN1*-KO NCI-H460 cells. Information for target exons is shown in the left panel. Blue box, constitutive exon; red boxes, skipped exon. Quantification of short splicing isoforms of the indicated SE genes from three independent biological replicates (top) and representative RT‒PCR images are shown (bottom). Data are represented as mean ± SD, analyzed by two-tailed unpaired *t* test; ***P* < 0.01; ****P* < 0.001; *****P* < 0.0001.

**
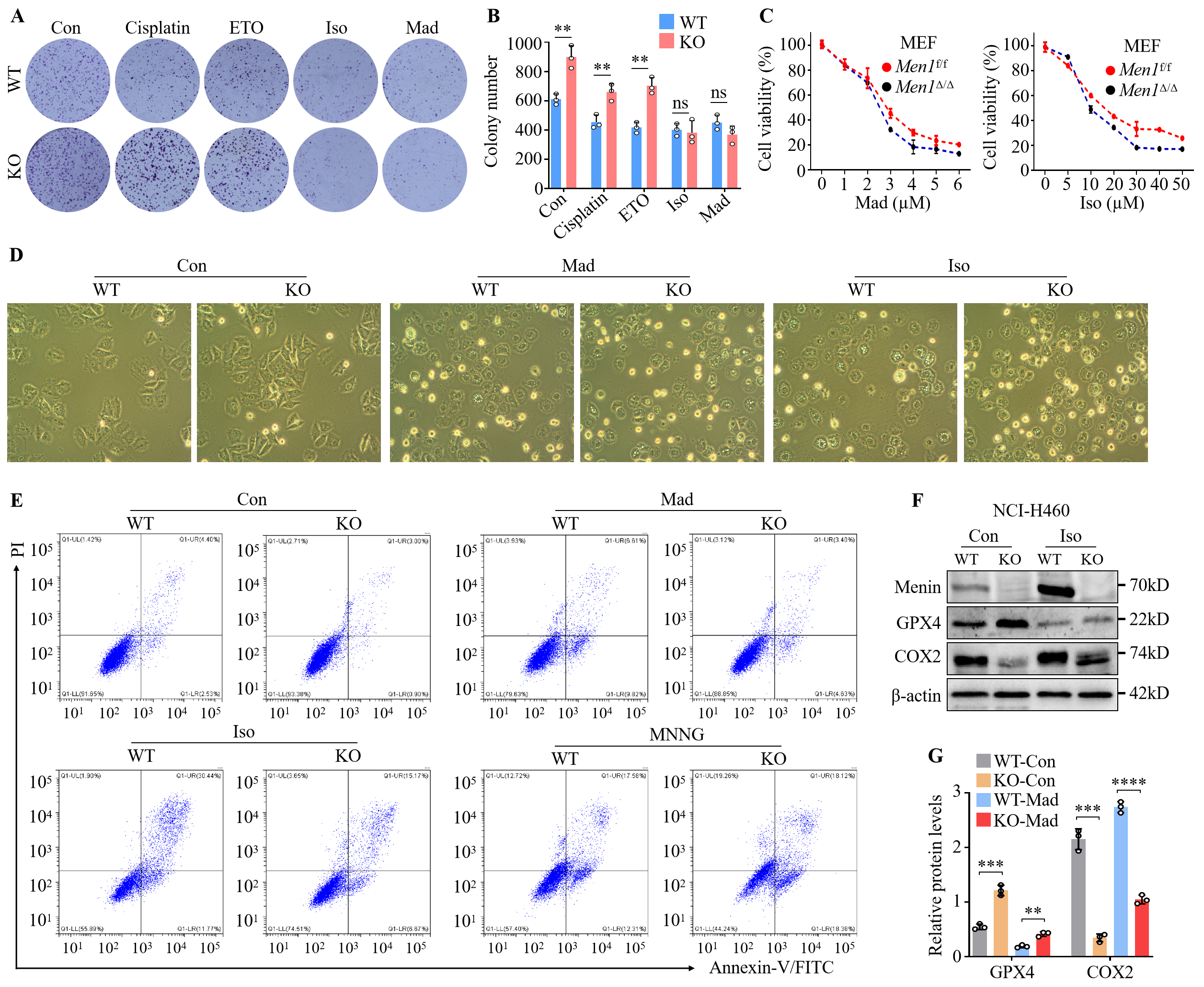
**

**Supplementary Figure 6. *MEN1* deficiency sensitizes human lung cancer cells to splicing inhibitors. (A, B)** Representative images of crystal violet staining (**A**) and quantification of colony number (**B**) in *MEN1*-WT and *MEN1*-KO NCI-H460 cells at 9 days after treatment with 2 μM Cisplatin, 0.2 mg/mL ETO, 2 μM Mad, or 20 μM Iso. **(C)** Relative cell viability of *Men1*^f/f^ and *Men1*^Δ/Δ^ MEFs under the indicated concentrations of Mad or Iso for 48 h. The relative cell viability in each group was normalized to the level of its untreated-group as 100%. **(D)** Representative morphological images of *MEN1*-WT and *MEN1*-KO NCI-H460 cells 48 h after treatment with 4 μM Mad or 40 μM Iso; scale bar, 40 μm. **(E)** Representative flow cytometry plots of Annexin-V and propidium iodide (PI) staining of *MEN1*-WT and *MEN1*-KO NCI-H460 cells following 48 h of 4 μM Mad, 40 μM Iso, 5 μM MNNG or DMSO (Con) treatment. **(F, G)** Immunoblotting of the indicated proteins (**F**) and quantification of relative protein levels (**G**) in *MEN1*-WT and *MEN1*-KO NCI-H460 cells treated with 20 μM Iso for 48 h. Data are represented as the mean ± SD (n = 3 biologically independent experiments), analyzed by two-tailed unpaired *t* test; ***P* < 0.01; ****P* < 0.001; *****P* < 0.0001; ns, not significant.

## Supplementary Table S1: Correlation analysis of menin expression and clinicopathological features in human lung cancer

| **Group** | **Cases** | **Menin expression (COD, SEM)** | ***P* value** |
| --- | --- | --- | --- |
| **Age (years)** |  |  | ns |
| ≦ 59.9 | 26 | 29648.22 ± 3085.54 |  |
| > 59.9 | 26 | 30984.24 ± 3643.55 |  |
| **Gender** |  |  | ns |
| Male | 27 | 30061.16 ± 3014.37 |  |
| Female | 25 | 30591.70 ± 3754.62 |  |
| **Tumor types** |  |  | ns |
| Adenocarcinoma | 42 | 30928.07 ± 2860.91 |  |
| Squamous carcinoma | 10 | 28035.74 ± 3612.86 |  |
| **Smoking history** |  |  | ns |
| No | 28 | 30708.01 ± 2947.99 |  |
| Yes | 24 | 29859.16 ± 3568.59 |  |
| **Tumor size** |  |  |  |
| 1 < T1 ≤ 2 cm | 25 | 39362.78 ± 3678.98 | T1 *vs* T2, *P* = 0.0086* |
| 2 < T2 ≤ 3 cm | 15 | 24823.37 ± 2820.96 | T1 *vs* T3, *P* = 0.0457* |
| 3 < T3 ≤ 4 cm | 6 | 23103.58 ± 3596.62 | T1 *vs* T4, *P* = 0.0022* |
| 4 < T4 ≤ 7 cm | 6 | 13567.07 ± 2356.54 | T3 *vs* T4, *P* = 0.0509 |
| **Stage** |  |  |  |
| I | 30 | 35779.54 ± 3448.91 | I *vs* II, *P* = 0.0710 |
| II | 9 | 23411.86 ± 3710.94 | I *vs* III, *P* = 0.0553 |
| III | 9 | 22456.43 ± 1083.18 | I *vs* IV, *P* = 0.1793 |
| IV/MIA | 4 | 22560.77 ± 3156.10 |  |

Data are represented as the mean ± SEM. Significance determined by ordinary one-way ANOVA; **P* < 0.05; ns, not significant; COD, corrected optical density

**Supplementary Table S2. The antibodies used in the article**

| **Antibodies** | **Isotype** | **Manufacturer** | **Cat.#** |
| --- | --- | --- | --- |
| Menin | Rabbit | Bethyl Laboratories | A300-105A |
| Menin antibody-ChIP Grade | Rabbit | Abcam | ab31902 |
| IgG | Rabbit | Cell Signaling | 2729 |
| β-actin | Mouse | Proteintech | 66009-1-Ig |
| Histone H3 | Rabbit | Cell Signaling | 4620 |
| MLL1 | Rabbit | Santa Cruz | sc-374392 |
| SNRPA | Rabbit | Proteintech | 10212-1-AP |
| SF3B2 | Rabbit | Proteintech | 10919-1-AP |
| U2AF65 | Mouse | SANTA CRUZ | sc-53942 |
| PRPF4 | Rabbit | Proteintech | 10728-1-AP |
| SNRNP200 | Rabbit | Proteintech | 23875-1-AP |
| hnRNPA1 | Rabbit | Abcam | ab177152 |
| Anti-RNA polymerase II | Rabbit | Abcam | ab264350 |
| Anti-RNA polymerase II CTD (phospho Ser 2) | Rabbit | Abcam | ab5095 |
| Phospho-Rbp1 CTD (Ser 5) | Rabbit | Cell Signaling | 2629 |
| Rbp1 CTD (4H8) | Mouse | Cell Signaling | 13523 |
| LEDGF | Rabbit | Bethyl Laboratories | A300-847A |
| Flag-Tag | Mouse | Absin | abs137958 |
| S9.6 | Mouse | Millipore | MABE1095 |
| 53BP1 | Rabbit | NOVUS | NB-100-305 |
| γH2AX (Ser139) | Mouse | Cell Signaling | 80312 |
| RNase H1 | Rabbit | Proteintech | 15606-1-AP |
| SRSF2 (SC35) | Mouse | Abcam | ab11826 |
| DDX17 | Rabbit | Abcam | ab24601 |
| MBNL1 | Mouse | Proteintech | 66837-1-1g |
| Phosoho-ATM (Thr1981) | Rabbit | Abcam | ab81292 |
| GPX4 | Mouse | Proteintech | 67763-1-Ig |
| COX2 | Rabbit | Proteintech | 27308-1-AP |
| Bcl-2 | Mouse | Proteintech | 68103-1-Ig |
| Caspase3 | Rabbit | Abcam | ab13847 |
| Ki67 | Rabbit | Cell Signaling | 9129 |
| HRP conjugate Goat Anti-Mouse IgG(H+L) | Mouse | Servicebio | GB23301 |
| HRP conjugate Goat Anti-Rabbit IgG(H+L) | Rabbit | Servicebio | GB23303 |
| FITC-conjugated Affinipure Donkey Anti-Rabbit IgG | Rabbit | Proteintech | SA00003-8 |
| Goat anti-Mouse IgG (H+L) Cross-Adsorbed Secondary Antibody, Alexa Fluor™ 594 | Mouse | Thermo Fisher Scientific | A11005 |

**Supplementary Table S3. The primers, siRNA, cell lines, plasmids, chemicals, mice, and human lung samples used in the article**

| **RT-PCR** |  | **Sequence (5’-3’)** |
| --- | --- | --- |
| M-*β-actin* | Forward | GCTGTGCTATGTTGCTCTAG |
|  | Reverse | GCTCGTTGCCAATAGTGATG |
| M-*Rbm27* | Forward | CACCAGTGGGAGCAAGAC |
|  | Reverse | GATGTGAGGCCAATCAGG |
| M-*Polm* | Forward | GGGTAGGGACTTACTGTGGC |
|  | Reverse | CATCGACGCTGAGACTGG |
| M-*Fance* | Forward | GAGACGTTCCTGGTGTTGC |
|  | Reverse | GAGACGTTCCTGGTGTTGC |
| M-*Prpf39* | Forward | ATCTTGTGCCCTCTATGA |
|  | Reverse | AGACATGCCTCACTCCTT |
| M-*Prpf40b* | Forward | AGCTGCTCTGAGGCTCTGGC |
|  | Reverse | GGGAATGGCTGGTGGTCT |
| M-*CD44* | Forward | GGAGATCAGGATGACTCCTTCT |
|  | Reverse | AGTCCTTGGATGAGTCTCGATC |
| H-*β-actin* | Forward | TCAGAAGGATTCCTATGTGGGCGA |
|  | Reverse | TTTCTCCATGTCGTCCCAGTTGGT |
| H-*MYL6* | Forward | GGACCAGGGCACCTATGA |
|  | Reverse | CAGGGAAAGGCACGGACT |
| H-*SLC4A7* | Forward | CTGGGTGAGACTCCTAAAG |
|  | Reverse | CAAATAAGGGCTGCAAAA |
| H-*SENP6* | Forward | CTGCGGCGTCTACCCTCC |
|  | Reverse | CAGAATCCTCATCTTCATCCAC |
| H-*GGPS1* | Forward | GGTGAAATAGTGGGAAGG |
|  | Reverse | GCTTGTCCTCTGGAACTT |
| H-*YTHDF3* | Forward | AAAGGGCAAGGAAATAAA |
|  | Reverse | TGACCAAGAAATGGAGGG |
| H-*EMSY* | Forward | GCAGAAACAGGAAGCAAG |
|  | Reverse | ATGTGGGGTGGTAAGGAG |
| H-*METTL6* | Forward | GGTTCTTAGGAGGCTTTA |
|  | Reverse | CCGCCAGAGTCTGTGGAT |
| H-*TFDP1* | Forward | GGATCTGGTAACATGGCAAAA |
|  | Reverse | GTTGACGGTGGAGGGGTG |
| H-*SLTM* | Forward | ATCACAGTGCTGATAGCG |
|  | Reverse | TTTTGGCTTACTTGCTTC |
| H-*CCNL2* | Forward | ATGATACCTTTCTAACAGC |
|  | Reverse | GAGACGCTGATCGGGATG |
| H-*CDH1* | Forward | ATCTTCAATCCCACCACG |
|  | Reverse | CTCGGACACTTCCACTCT |
| H-*ZNF18* | Forward | CTCAAGTCCCAACTCCTG |
|  | Reverse | GAATACCCTCCTCTTTGC |
| H-*CDK4* | Forward | CTCCCGACTCCTCCATCT |
|  | Reverse | TATGCAACACCTGTGGACAT |
| H-*CD44*-C3-5’ | Forward | AGGCTTCCTTCTGTTTGT |
|  | Reverse | TGGGAGGTGTTGGATGTG |
| H-*CD44*-C3-3’ | Forward | ATCCAACACCTCCCAGTA |
|  | Reverse | TTCTCCCAATGTGACCTA |
| H-*CD44*-C5-5’ | Forward | CTCAGAGTTGCCTTGTCC |
|  | Reverse | CATCATCAGTAGGGTTGC |
| H-*CD44*-C5-3’ | Forward | ACCCGCTATGTCCAGAAA |
|  | Reverse | ACAACCACAAGAGCCCAC |
| H-*CD44*-V5-5’ | Forward | TTTGTGGTTCTGCCTTTG |
|  | Reverse | GTGGTGCCATTTCTGTCT |
| H-*CD44*-V5-3’ | Forward | ATGTAGACAGAAATGGCACCAC |
|  | Reverse | CCTTTGGGCAACCTGATG |
| H-*CD44*-V6-5’ | Forward | CCTGTTTCCTTGGTGCCT |
|  | Reverse | CCTCATGCCATCTGTTGC |
| H-*CD44*-V6-3’ | Forward | CACCCAAAGAAGACTCCC |
|  | Reverse | CTCGTGACAATTCCTCCA |
| H-*CD44*-V8-5’ | Forward | GTTGTGGTGCTCTTTGAT |
|  | Reverse | AGTAGGCTGAAGCGTTAT |
| H-*CD44*-V8-3’ | Forward | CAGCCTACTGCAAATCCA |
|  | Reverse | GACCTCCACAAGAAATACAGAC |
| H-*CD44*-V10-5’ | Forward | AACTTCACTCTATTTCTCCCTA |
|  | Reverse | GGTCCTGCTTTCCTTCGT |
| H-*CD44*-V10-3’ | Forward | TCATCCCAGTGACCTCAG |
|  | Reverse | TCCATCATAACAGCCAAC |
| H-*FAM189B* | Forward | CCTCTTCCGTCACCACCAC |
|  | Reverse | GGCACAGGCGGGACAAAT |
| H-*NUBP2* | Forward | GCACATCATCCTGGTCCTCT |
|  | Reverse | CAGGGCTTCTATGGTGGC |
| H-*ENDOV* | Forward | AACGCTGTCACTGTGGAAAC |
|  | Reverse | ACACGGCAGGTCTGTAAGG |
| H-*TARBP2* | Forward | ATGAGTGAAGAGGAGCAAGGC |
|  | Reverse | CAGACTGCTGAGGGGAGACA |
| H-*CPSF7* | Forward | GACCCAGAGTTCAACAATAC |
|  | Reverse | TTCAGAGGCTACCACCAC |
| H-*MRRF* | Forward | TGCCTTGGTTGAGGATAT |
|  | Reverse | TGTTCATTGAGTTGGTGC |
| H-*LETMD1* | Forward | GGGAGATGGAGCATTTGA |
|  | Reverse | ATTCAGGCCACGGAGATA |
| **RT-qPCR** |  |  |
| H-*IRF1*-5′ | Forward | CTGCCAGATATCGAGGAGGTGAAA |
| H- *IRF1*-3′-intron | Reverse | GACCCAGAGTCCTTGGATACCTTT |
| H- *IRF1*-3′-exon | Reverse | TCTTGGCCTTGCTCTTAGCATCTC |
| H-*CD44*-V6（Fig. 2F） | Forward | GTAGACAGAAATGGCACCAC |
|  | Reverse | CAGCTGTCCCTGTTGTCGAA |
| H-*CD44*-V8（Fig. 2F） | Forward | CAGCTGTCCCTGTTGTCGAA |
|  | Reverse | GAGGTCCTGTCCTGTCCAAA |
| H-*CD44*-V10（Fig. 2F） | Forward | GGAATGATGTCACAGGTGGA |
|  | Reverse | AGGTCACTGGGATGAAGGTC |
| H-*CD44*-C2 | Forward | TGCCGCTTTGCAGGTGTATT |
|  | Reverse | GGCAAGGTGCTATTGAAAGCCT |
| H-*CD44*-C3 | Forward | ACAGGTATGGGTTCATAGAAGGGCAC |
|  | Reverse | TGTGTCATACTGGGAGGTGTTGGA |
| H-*CD44*-C5 | Forward | AGCAGCACTTCAGGAGGTTACA |
|  | Reverse | TGATCCAGGGACTGTCTTCGT |
| H-*CD44*-V2 | Forward | CAGCAACTGAGACAGCAACCAA |
|  | Reverse | AACCAATCCCAGGTTTCTTGCC |
| H-*CD44*-V3 | Forward | GGCTGGGAGCCAAATGAAGAAA |
|  | Reverse | CATCATCATCAATGCCTGATCCAGA |
| H-*CD44*-V4 | Forward | CAGTGGAACCCAAGCCATTCAA |
|  | Reverse | CCTTGTGGTTGTCTGAAGTAGCAC |
| H-*CD44*-V5 | Forward | GAAACTGGAACCCAGAAGCACA |
|  | Reverse | TGATGCTCATGGTGAATGAGGG |
| H-*CD44*-V6 | Forward | CAGAAGGAACAGTGGTTTGGCA |
|  | Reverse | GTCTTCTTTGGGTGTTTGGCGA |
| H-*CD44*-V7 | Forward | TGCAAGGAAGGACAACACCAAG |
|  | Reverse | GGGTGTGAGATTGGGTTGAAGA |
| H-*CD44*-V8 | Forward | ACGCTTCAGCCTACTGCAAA |
|  | Reverse | AAGAGGTCCTGTCCTGTCCAAA |
| H-*CD44*-V9 | Forward | GAGCTTCTCTACATCACATGAAGGC |
|  | Reverse | GTCAGAGTAGAAGTTGTTGGATGGTC |
| H-*CD44*-V10 | Forward | ACCTCTCATTACCCACACACGA |
|  | Reverse | TAGCTGAGGTCACTGGGATGAA |
| H-*CD44*-i15 | Forward | CCTGCCGCCAAGAAATCTCTGTTT |
|  | Reverse | TGCAAATGGGCATACTCTGGCT |
| H-*CD44*-i16 | Forward | TGGTCTGAGAGCCACACTTTGAGT |
|  | Reverse | TAACTAGCCATGTGTGCTGGGTCA |
| H-*CD44*-i18 | Forward | 5’-AGGGAAATGCAGGCCAACCAAA |
|  | Reverse | ATGGGTGGTTGAGGGATGGAATGA |
| H-*CD44*-C19 | Forward | TGATCAACAGTGGCAATGGAGC |
|  | Reverse | TCTGACGACTCCTTGTTCACCA |
| Utrophin-Ex.1/In.1 | Forward | GGCAAGATGGCCAAGTATGGAG |
|  | Reverse | GCTTTCTTGAGCTTCCTTTACCTACCAG |
| Utrophin-Ex.2/In.2 | Forward | CCATTCCACAGATGAACACAATGACG |
|  | Reverse | GCGGCATCTGAACCATCGAAGT |
| **ChIP-qPCR** |  |  |
| H-*CD44*-PP | Forward | TCCCTCCGTCTTAGGTCACTGTTT |
|  | Reverse | CCTCGGAAGTTGGCTGCAGTTT |
| H-*CD44*-C1 | Forward | CTCCGGACACCATGGACAAGTTT |
|  | Reverse | CCAATGCACTTCGCAGACAGCTCA |
| H-*CD44*-i1 | Forward | TGCGCTAAACCGTTGGAGAA |
|  | Reverse | CGGTTACTTTCTGCCATGTGCT |
| H-*CD44*-C2 | Forward | TGCCGCTTTGCAGGTGTATT |
|  | Reverse | GGCAAGGTGCTATTGAAAGCCT |
| H-*CD44*-C3 | Forward | ACAGGTATGGGTTCATAGAAGGGCAC |
|  | Reverse | TGTGTCATACTGGGAGGTGTTGGA |
| H-*CD44*-C4 | Forward | ACATCAGTCACAGACCTGCCCAAT |
|  | Reverse | AACACACCTGAGCCCTTTCCC |
| H-*CD44*-C5 | Forward | AGCAGCACTTCAGGAGGTTACA |
|  | Reverse | TGATCCAGGGACTGTCTTCGT |
| H-*CD44*-V2 | Forward | CAGCAACTGAGACAGCAACCAA |
|  | Reverse | AACCAATCCCAGGTTTCTTGCC |
| H-*CD44*-V3 | Forward | GGCTGGGAGCCAAATGAAGAAA |
|  | Reverse | CATCATCATCAATGCCTGATCCAGA |
| H-*CD44*-V4 | Forward | CAGTGGAACCCAAGCCATTCAA |
|  | Reverse | CCTTGTGGTTGTCTGAAGTAGCAC |
| H-*CD44*-V5 | Forward | GAAACTGGAACCCAGAAGCACA |
|  | Reverse | TGATGCTCATGGTGAATGAGGG |
| H-*CD44*-V6 | Forward | CAGAAGGAACAGTGGTTTGGCA |
|  | Reverse | GTCTTCTTTGGGTGTTTGGCGA |
| H-*CD44*-V7 | Forward | TGCAAGGAAGGACAACACCAAG |
|  | Reverse | GGGTGTGAGATTGGGTTGAAGA |
| H-*CD44*-V8 | Forward | ACGCTTCAGCCTACTGCAAA |
|  | Reverse | AAGAGGTCCTGTCCTGTCCAAA |
| H-*CD44*-V9 | Forward | GAGCTTCTCTACATCACATGAAGGC |
|  | Reverse | GTCAGAGTAGAAGTTGTTGGATGGTC |
| H-*CD44*-V10 | Forward | ACCTCTCATTACCCACACACGA |
|  | Reverse | TAGCTGAGGTCACTGGGATGAA |
| H-*CD44*-i15 | Forward | CCTGCCGCCAAGAAATCTCTGTTT |
|  | Reverse | TGCAAATGGGCATACTCTGGCT |
| H-*CD44*-C16 | Forward | TGCAGTACTGACCTTCCTGATTGCTC |
|  | Reverse | GGTGGAATGTGTCTTGGTCTCCTGTA |
| H-*CD44*-i16 | Forward | TGGTCTGAGAGCCACACTTTGAGT |
|  | Reverse | TAACTAGCCATGTGTGCTGGGTCA |
| H-*CD44*-C18 | Forward | TGTCTCTGAAGCTCACGCATGTCA |
|  | Reverse | CGACTGTTGACTGCAATGCAAACTGC |
| H-*CD44*-i18 | Forward | AGGGAAATGCAGGCCAACCAAA |
|  | Reverse | ATGGGTGGTTGAGGGATGGAATGA |
| H-*CD44*-C19 | Forward | TGATCAACAGTGGCAATGGAGC |
|  | Reverse | TCTGACGACTCCTTGTTCACCA |
| H-*c-Myc* -2000 | Forward | AAGACGCTTTGCAGCAAAATC |
|  | Reverse | AGGCCTTTGCCGCAAAC |
| H-*c-Myc* -200 | Forward | GTAGTTAATTCATGCGGCTCTCTTACT |
|  | Reverse | GGGCAGCCGAGCACTCTA |
| H-*c-Myc* 1 | Forward | GGAGGGATCGCGCTGAGTA |
|  | Reverse | TCTGCCTCTCGCTGGAATTAC |
| H-*c-Myc* 190 | Forward | GCCGCATCCACGAAACTTT |
|  | Reverse | TCCTTGCTCGGGTGTTGTAAG |
| H-*c-Myc* 393 | Forward | CGGGTAGTGGAAAACCAGGTAA |
|  | Reverse | TCGACTCATCTCAGCATTAAAGTGA |
| H-*c-Myc* 593 | Forward | GATGGGAGAGGAGAAGGCAGA |
|  | Reverse | GGCAGAAATCTCGAAAGGGTAG |
| H-*c-Myc* 2018 | Forward | TGCCCCTCAACGTTAGCTTC |
|  | Reverse | GGCTGCACCGAGTCGTAGTC |
| H-*c-Myc* 2789 | Forward | CGGCTGGATACCTTTCCCAT |
|  | Reverse | TTCCTAATAAGAGTGGCCCGTTAA |
| H-*c-Myc* 3389 | Forward | GGATCTTCTCAGCCTATTTTGAACA |
|  | Reverse | TTGCAGTTTTGCCAAAAGTCC |
| H-*c-Myc* 3589 | Forward | GTCCAAAGCCTCATTAAGTCTTAGGTA |
|  | Reverse | CAACTTCCCAGGATAGGACATTG |
| H-*c-Myc* 4828 | Forward | CCTGAGCAATCACCTATGAACTTG |
|  | Reverse | CAAGGTTGTGAGGTTGCATTTG |
| H-*c-Myc* 5071 | Forward | ACACAATGTTTCTCTGTAAATATTGCCA |
|  | Reverse | ACTAGGATTGAAATTCTGTGTAACTGCT |
| H-*c-Myc* 5155 | Forward | AAGTACATTTTGCTTTTTAAAGTTGATT |
|  | Reverse | GGCTCAATGATATATTTGCCAGTTATTTTA |
| H-*c-Myc* 5428 | Forward | GATGCTTCCTGGAGACTATGATAACA |
|  | Reverse | GCCTTCTGCCATTCCTTCTAACT |
| H-*c-Myc* 6028 | Forward | TCCCCATATTAGAAGTAGAGAGGGAA |
|  | Reverse | CTTGGGCATGTGGATGAGTCT |
| H-*c-Myc* 7028 | Forward | ATCGGGAAGGTGTTAGTCTGAATC |
|  | Reverse | CACTCTCTCCTATTCTGAGGGCTT |
| H-*c-Myc* *11382* | Forward | TGAAGGATGTTGGAGCATGAGTA |
|  | Reverse | TCTCTTCCCAGTTGAGTCTTGAAA |
| **DRIP-qPCR** |  |  |
| H-*CALM3* | Forward | GAGGAATTGTGGCGTTGACT |
|  | Reverse | AGAGTGGCCAAATGAGCAGT |
| H-*TFPT* | Forward | TCTGGGAGTCCAAGCAGACT |
|  | Reverse | AAGGAGCCACTGAAGGGTTT |
| H-*PRMT2* | Forward | GCATTAGCGCCACCCATTT |
|  | Reverse | GGAAGCAGAATGATGACGTTTCT |
| H-*MYC* | Forward | CACTTTGCACTGGAACTTACAACA |
|  | Reverse | TCCCCAAATGGGCAGAATAG |
| H-*rDNA* | Forward | GGTATATCTTTCGCTCCGAGTC |
|  | Reverse | GGACAGCGTGTCAGCAATAA |
| H-*TM4SF1* | Forward | TGGTTGAGGCTTTGAAAGACAGT |
|  | Reverse | CAGATTGGAAGCTGTCCAGACA |
| **Cell lines** |  |  |
| Mouse: MEFs | This paper | N/A |
| Human: NSCLC A549 | ATCC | CRL-2190 |
| Human: SCLC NCI-H460 | This paper | 631530 |
| Human: LCLC NCI-H446 | ATCC | CRL-1573 |
| Human: HEK-293T | This paper | N/A |
| **Lung cancer samples** |  |  |
| Adenocarcinoma samples (n = 42) | Department of thoracic surgery, Affiliated Hospital of Guizhou Medical University | N/A |
| Squamous carcinoma samples (n = 8) |  | N/A |
| Lung neuroendocrine carcinoma samples (n = 2) |  | N/A |
| **Mice/Organisms/Strains** |  |  |
| *Men1*^f/f^:129S(FVB)-*Men1*^tm1.2Ctre^/J mouse | Jackson Laboratory | 005109 |
| *Ubc*-Cre: B6;129S-Tg(*UBC*-cre/ERT2)1Ejb/J C57BL/6 mouse | Jackson Laboratory | 007001 |
| BALB/c nude mouse | Beijing HFK Bioscience CO.,Ltd | N/A |
| **Plasmids and virus strains** |  |  |
| pLVX-CMV-*MEN1* plasimd | This paper | N/A |
| pLVX-CMV-*RNase H1* plasimd | This paper | N/A |
| pCDH-CMV-sh*MEN1* plasimd | This paper | N/A |
| pSpCas9-2A-GFP (PX458) vector | 48138 | Addgene |
| pSpCas9-2A-Puro (PX459) vector | 48139 | Addgene |
| **Chemicals, drugs, and Kits** |  |  |
| Corn oil | C8267 | Sigma |
| Total RNA Extraction Reagent | 10606ES60 | Yeasen |
| Hifair ^®^Ⅲ 1st strand cDNA Synthesis SuperMIX for qPCR | 11141ES60 | Yeasen |
| TB Green^®^ Premix Ex Taq^TM^Ⅱ | RR820A | TaKaRa |
| Ethidium Bromide dye | C14141868 | MACKLIN |
| RNase inhibitor | R8061 | Solarbio |
| Protease inhibitor cocktail | P6730 | Solarbio |
| Digitonin | 11024-24-1 | Sigma |
| DNase I | 9003-98-9 | Merck |
| Spermidine | 05292-1ML-F | Sigma |
| EcoRI | ER0271 | Thermo Fisher Scientific |
| HindIII | ER0502 | Thermo Fisher Scientific |
| Xbal | ER0681 | Thermo Fisher Scientific |
| Sspl | ER0771 | Thermo Fisher Scientific |
| BsrGI | ER0931 | Thermo Fisher Scientific |
| Tris-saturated Phenol | C14640130 | MACKLIN |
| 3-Methyl-1-butanol | C14554758 | MACKLIN |
| Glycogen | 10901393001 | Roche |
| Chemi-Trans^TM^ FectinBor DNA Transfection Reagent | T008 | GeneCodex |
| Micrococcal nuclease | 10011 | Cell Signaling |
| RIPA lysis buffer | P0013B | Beyotime |
| RNase H | M0297S | New England Biolabs |
| Methylene Blue Solution | G1303 | Solarbio |
| NP-40 lysis buffer | ST2045 | Beyotime |
| Crystal Violet Stain solution | G1062 | Solarbio |
| Tamoxifen | T5648-1G | Sigma |
| Phorbol ester PMA | 16561-29-8 | MCE |
| MI-3 | 1271738-59-0 | MCE |
| Cisplatin | 15663-271 | Solarbio |
| Etoposide | IE0270 | Solarbio |
| Mitomycin C | 924-16-3 | Sigma |
| Isoginkgetin | GC12353 | GLPBIO |
| Madrasin | HY-100236 | MCE |
| MNNG | HY-N2117 | MCE |
| Actinomycin D | GC16866 | GLPBIO |
| DRB | C4798 | APEXBIO |
| Aphidicolin | HY-N6733 | MCE |
| Hoechst 33342 | B2261 | Sigma |
| Mycoplasma Stain Assay Kit | C0296 | Beyotime |
| RNAprep Pure Kit | DP432 | TIANGEN Biotech |
| Simple ChIP Kit | 9003S | Cell Signaling |
| Cell Counting Kit-8 | BS350 | Biosharp |
| Annexin V-FITC/PI Apoptosis Detection Kit | 40302ES60 | Yeasen |
| TIANamp Genomic DNA Kit | DP304-03 | TIANGEN |
| BeyoClick^TM^ EdU Cell Proliferation Kit with Aleaxa Fluor 488 | C0071S | Beyotime |
